# Decoding of translation‐regulating entities reveals heterogeneous translation deficiency patterns in cellular senescence

**DOI:** 10.1111/acel.13893

**Published:** 2023-08-07

**Authors:** Angelos Papaspyropoulos, Orsalia Hazapis, Abdullah Altulea, Aikaterini Polyzou, Panayotis Verginis, Konstantinos Evangelou, Maria Fousteri, Argyris Papantonis, Marco Demaria, Vassilis Gorgoulis

**Affiliations:** ^1^ Molecular Carcinogenesis Group, Department of Histology and Embryology, School of Medicine National Kapodistrian University of Athens (NKUA) Athens Greece; ^2^ Biomedical Research Foundation Academy of Athens Athens Greece; ^3^ European Research Institute for the Biology of Ageing (ERIBA) University Medical Center Groningen Groningen The Netherlands; ^4^ Medical School University of Crete Heraklion Greece; ^5^ Institute for Fundamental Biomedical Research Biomedical Sciences Research Center “Alexander Fleming” Vari Greece; ^6^ Institute of Pathology University Medical Center Göttingen Göttingen Germany; ^7^ Center for Molecular Medicine Cologne University of Cologne Cologne Germany; ^8^ Clinical Molecular Pathology Medical School, University of Dundee Dundee UK; ^9^ Molecular and Clinical Cancer Sciences, Manchester Cancer Research Centre, Manchester Academic Health Sciences Centre University of Manchester Manchester UK; ^10^ Center for New Biotechnologies and Precision Medicine Medical School, National and Kapodistrian University of Athens Athens Greece; ^11^ Faculty of Health and Medical Sciences University of Surrey Surrey UK

**Keywords:** IRES elements, oncogene‐induced senescence, replicative senescence, ribosome stalling, stress‐induced senescence, translation deficiency, uORF/dORF

## Abstract

Cellular senescence constitutes a generally irreversible proliferation barrier, accompanied by macromolecular damage and metabolic rewiring. Several senescence types have been identified based on the initiating stimulus, such as replicative (RS), stress‐induced (SIS) and oncogene‐induced senescence (OIS). These senescence subtypes are heterogeneous and often develop subset‐specific phenotypes. Reduced protein synthesis is considered a senescence hallmark, but whether this trait pertains to various senescence subtypes and if distinct molecular mechanisms are involved remain largely unknown. Here, we analyze large published or experimentally produced RNA‐seq and Ribo‐seq datasets to determine whether major translation‐regulating entities such as ribosome stalling, the presence of uORFs/dORFs and IRES elements may differentially contribute to translation deficiency in senescence subsets. We show that translation‐regulating mechanisms may not be directly relevant to RS, however uORFs are significantly enriched in SIS. Interestingly, ribosome stalling, uORF/dORF patterns and IRES elements comprise predominant mechanisms upon OIS, strongly correlating with Notch pathway activation. Our study provides for the first time evidence that major translation dysregulation mechanisms/patterns occur during cellular senescence, but at different rates depending on the stimulus type. The degree at which those mechanisms accumulate directly correlates with translation deficiency levels. Our thorough analysis contributes to elucidating crucial and so far unknown differences in the translation machinery between senescence subsets.

## Graphical abstract

Stress‐induced senescence, in contrast to replicative senescence, is accompanied by distinct translation deregulation mechanisms. Oxidative stress relies mostly on uORFs, whereas oncogenic stress employs ribosome stalling, uORFs/dORFs and IRES elements to lead to macromolecular damage, a key hallmark of senescence.

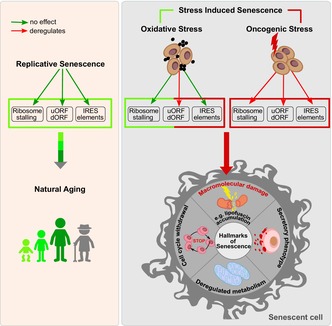

**Abbreviations**
3′UTR: 3′ untranslated region
5′ TOP motifs: 5′ Terminal oligopyrimidine motifs
5′UTR: 5′ untranslated region
A‐site: ribosome aminoacylsite
APA: alternative polyadenylation
CDF: cumulative distribution function
CDS: coding sequence
DDR: DNA damage response
DMS: dimethyl sulfate sequencing
dORF: downstream open reading frame
E‐site: ribosome exit site
EOIS: escape from oncogene‐induced senescence
FDR: false discovery rate
GSEA: gene set enrichment analysis
IRES: internal ribosome entry sites
OIS: oncogene‐induced senescence
ORF: open reading frame
OSIS: oxidative stress‐induced senescence
P‐site: ribosome peptidyl site
ROS: reactive oxygen species
RS: replicative senescence
SASP: senescence‐associated secretory phenotype
SHAPE‐seq: selective 2′‐hydroxyl acylation analyzed by primer extension and sequencing
SIS: stress‐induced senescence
uORF: upstream open reading frame

## 1 INTRODUCTION

Cellular senescence is characterized by a prolonged and generally irreversible cell cycle arrest, accompanied by macromolecular damage, distinct metabolic features and a unique secretome collectively referred to as Senescence‐Associated Secretory Phenotype (SASP) (Gorgoulis et al., 2019). Cellular senescence is often the outcome of age‐related diseases, but it is not synonymous to aging. Indeed, senescence programs can be triggered by a plethora of stressful insults such as exposure to genotoxic agents and oncogene activation, regardless of organismal age (Casella et al., 2019; Shay et al., 1991). For this reason, senescence frequently constitutes a potent anti‐tumorigenic barrier (Braig et al., 2005; Chen et al., 2005; Collado et al., 2005; Kang et al., 2011; Michaloglou et al., 2005).

Several types of cellular senescence have been identified. Replicative senescence (RS) is the natural outcome of multiple cell division rounds leading to progressive telomere attrition, which in turn leads to activation of DNA damage response (DDR) pathways (d'Adda di Fagagna et al., 2003; Shay & Wright, 2019). Stress‐induced senescence (SIS) is activated in response to acute stress insults triggering the DDR (Chen & Ames, 1994; Hewitt et al., 2012), and oncogene‐induced senescence (OIS) is a subtype of SIS elicited by oncogene activation (Serrano et al., 1997). The various senescence types display distinct molecular features depending on the nature of the stimulus and cell type (Casella et al., 2019).

Macromolecular damage at the protein level is a feature of cellular senescence (Sabath et al., 2020). For instance, senescent cells display elevated levels of lipofuscin, one of the major senescence hallmarks (Georgakopoulou et al., 2013). Lipofuscin is a non‐degradable byproduct of the senescence process comprised of aggregates of oxidized proteins, lipids, and metals (Georgakopoulou et al., 2013), and those aggregates may partly be the outcome of overproduction or defective degradation of individual components. Translation deficiency may additionally constitute one of the factors contributing to protein aggregates found in lipofuscin. Indeed, an overall reduction of protein synthesis accompanied by inhibition of ribosome biogenesis has been reported in senescent cells (Lessard et al., 2018; Nishimura et al., 2015).

Protein translation in eukaryotes is vastly dependent on the presence of fully functional ribosomes and initiates when Eukaryotic Initiation Factor 2 (EIf2) elicits the binding of methionine (Met) tRNA to the ribosome in a GTP‐dependent manner (Trachsel & Staehelin, 1978). In order to identify the first ATG codon (start codon), the ribosome scans the mRNA for the characteristic Kozak motif surrounding the codon (Kozak, 1986). Upon recognition of the start codon, the large and small ribosomal subunits combine to form the 80S subunit which initiates high fidelity translation (Hernandez et al., 2019). However, several aspects along this process may contribute to premature termination of translation and rapid mRNA decay such as “No‐Go Decay” where mRNAs containing stalled ribosomes are degraded (Doma & Parker, 2006; Harigaya & Parker, 2010). Colliding ribosomes or ribosome stalling may occur when the ribosome comes across truncated RNA or a polyA region on the coding DNA sequence (CDS) or even upon encountering “slippery codons” which cause ribosome frameshifting and, thus, alterations in the rate of translation or lead to defective protein products (Clark et al., 2007; Meydan & Guydosh, 2020).

Although translation normally begins at the ATG start codon of the CDS, on certain occasions it was also found to initiate from other sites toward the 5′ untranslated region (5′ UTR) upstream of the CDS or the 3′ untranslated region (3′ UTR) downstream the termination codons (Chew et al., 2016; Johnstone et al., 2016; Wu et al., 2020). Those ~100 nucleotide (nt)‐long upstream or downstream open reading frames (ORFs) are known as uORFs or dORFs, respectively, and may overlap with CDS. It has been shown that uORFs may suppress translation of their downstream canonical ORFs, while dORFs may enhance translation of their upstream canonical ORFs; however, the exact mechanism behind this regulation remains unknown (Chew et al., 2016; Johnstone et al., 2016; Wu et al., 2020). Recent mass‐spectrometry evidence has revealed that those small ORFs may encode peptides holding potential regulatory functions (Jayaram et al., 2021).

On the other hand, Internal Ribosome Entry Sites (IRES) are RNA elements which may affect the selection of start codons for ORFs, thereby indirectly contributing to deregulation of canonical ORF translation (Hentze et al., 1988; Spriggs et al., 2008; Stoneley et al., 1998). IRES elements are mostly found in genes involved in stress, where they facilitate cap‐independent mRNA translation (Spriggs et al., 2008). Thus, in addition to ribosome stalling, uORFs/dORFs, aided by respective IRES elements, may act as functional switches controlling the translation efficiency of canonical ORFs.

Although translation deficiency has been identified in senescent cells, the exact mechanisms contributing to it upon different insults or through natural processes remain elusive. In this study, we interrogate large published RNA sequencing and ribosome profiling datasets, complemented by subsequent experimental confirmation, to identify distinct patterns of translation deregulation in different types of senescence. We provide evidence of senescence subset‐specific differential disruptions of the translation machinery, thereby explaining how the expression of key molecular players and the activity of signaling pathways may be mechanistically modulated at the ribosome/mRNA level depending on the senescence type. Additionally, our findings explain how translation deregulation mechanisms may lead to lipofuscin accumulation, a fundamental characteristic of senescence.

## 2 RESULTS

To investigate the most prominent translation defects, we first analyzed published data from human cell lines and mouse models pertaining to cells where senescence was induced by different stimuli (Table S1). We focused on ribosome profiling and RNA‐seq datasets allowing for the identification of ribosome stalling, uORFs, dORFs and IRES elements as key translation deregulation patterns (Figure S1). Our pipeline included several filtering and quality control steps in order to determine ribosome periodicity and calculate P‐site offset and coverage by using the RiboTaper pipeline (Calviello et al., 2016). In parallel, RNA‐seq data were used to normalize ribosome fragments, evaluate translation efficiency changes and estimate the alternative splicing patterns that potentially impact translation, such as having truncated reads over the CDS. RNA‐seq data were also implemented in examining alternative polyadenylation (APA) events which may result in ribosome stalling due to the repetition of premature polyA signals among the last exons (Arthur et al., 2015). Along those lines, we additionally explored how major signaling pathway components were affected by the identified translation defects (Figure S1).

### 2.1 Replicative senescence is not accompanied by distinct translation deregulation patterns

**FIGURE 1:**
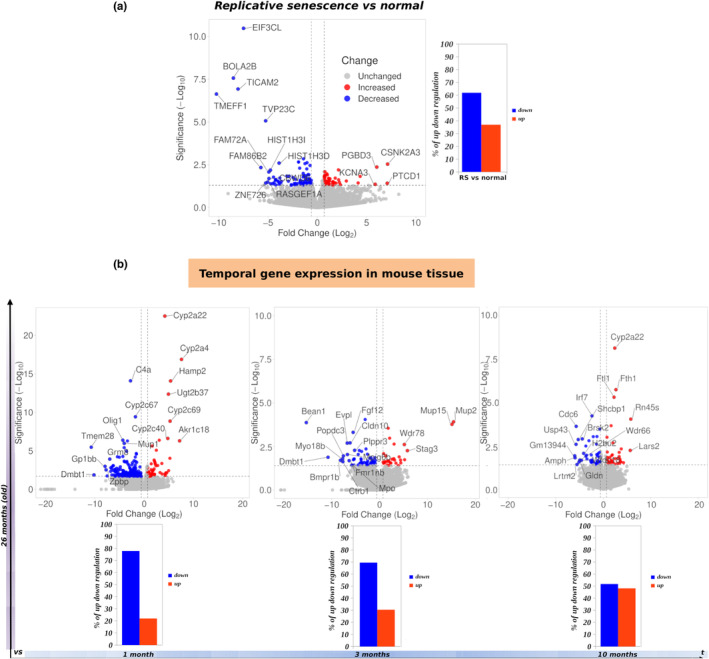
Replicative senescence is accompanied by translation deficiency in vitro and in vivo. (a) Volcano plots demonstrating transcripts with significantly decreased (blue) and increased (red) translation rate in human W138 lung fibroblasts undergoing RS versus control. Bar graphs indicate the percentage of significant (*p* < 0.05) changes in translation efficiency. See also Table S2. (b) Same as (a) for aged (26 months old) mouse liver versus liver tissue from younger mice, whose age is displayed incrementally on the *t* axis. Translation deficiency changes are progressively diminished as 26‐month‐old mice are compared with mice approaching their age. See also Figure S2A–C.

To uncover potential translation deregulation patterns in RS, we first implemented RNA‐seq and Ribo‐seq data derived from human W138 fibroblasts which were passaged until they reached RS (Sabath et al., 2020). Senescent WI38 cells were compared to young, non‐senescent counterparts. In line with previous observations (Gonskikh & Polacek, 2017; Sabath et al., 2020), we found that RS cells displayed an increased level of translation deficiency compared to controls (Figure 1a). To validate the above observation using data from in vivo studies, we utilized a combination of Ribo‐seq and RNA‐seq data collected throughout the entire lifespan of mice, focusing on liver and kidney tissue (Anisimova et al., 2020). We found that cells from aged tissues with upregulated senescence markers (Anisimova et al., 2020) concurrently displayed reduced translation efficiency compared to young mice (Figure 1b). Particularly, our data confirmed that translation efficiency was proportionally decreased with age in both tissues (Figure 1b and Figure S2A–C).

**FIGURE 2:**
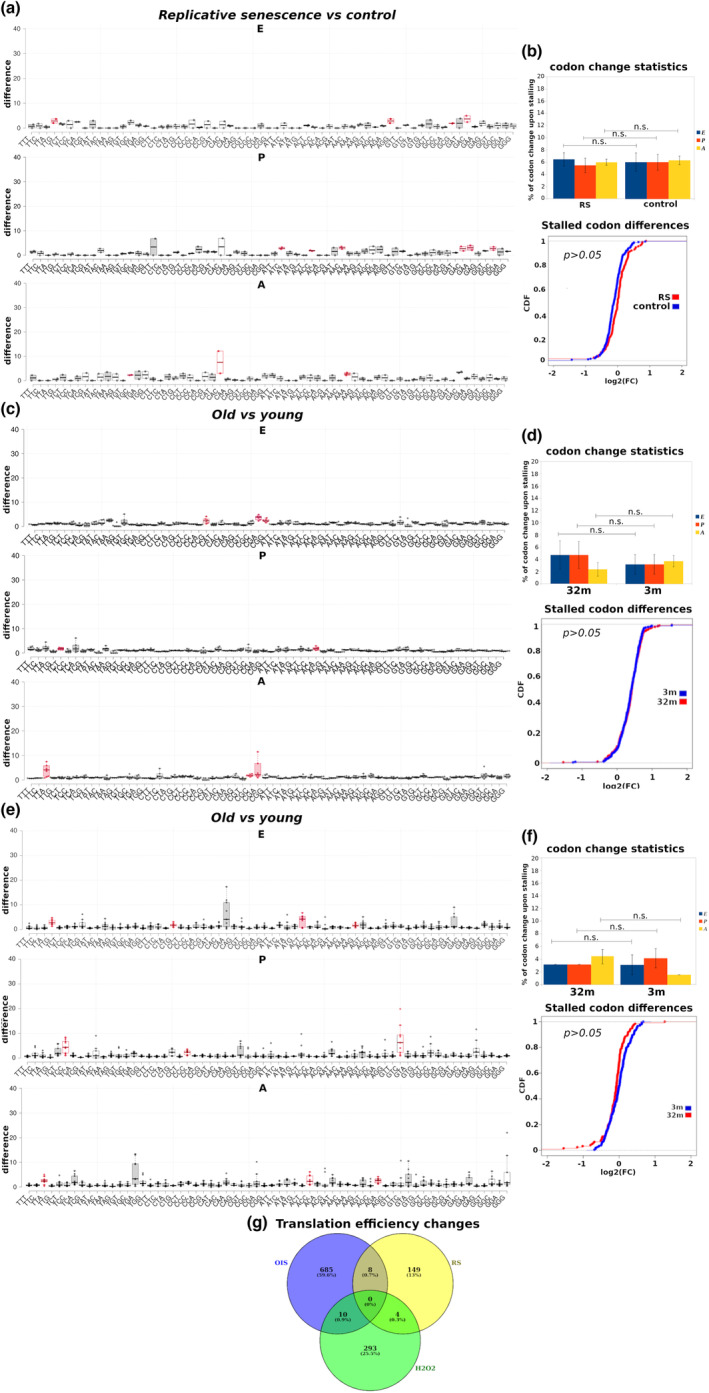
Replicative senescence displays no distinct translation deficiency patterns. (a) Ribosome stalling in E‐, P‐ or A‐ sites in human W138 lung fibroblasts under RS versus control. Red coloring in the box plot (left) indicates codons where ribosomes are most stalled. (b) Top: The bar graphs indicate a non‐significant overall difference in the percentage of stalled codons per library between RS and control. Bottom: Cumulative distribution function (CDF) curve showing non‐significant differences in translation efficiency between stalled codons in RS versus control cells. See also Table S2. (c) Same as (a), for aged (32 months old) mouse kidney tissue versus respective young tissue (3 months old). (d) Top: No significant overall differences in the percentage of stalled codons per library were identified. See also Figure S3B. Bottom: CDF curve showing no significant differences in translation efficiency between stalled codons in 32‐month versus 3‐month mouse kidney cells. (e) Same as (a), for aged (32 months old) mouse liver tissue versus respective young tissue (3 months old). (f) Top: No significant overall differences in the percentage of stalled codons per library were identified. See also Figure S3C. Bottom: CDF curve showing no significant differences in translation efficiency between stalled codons in 32‐month versus 3‐month mouse liver cells. (g) Venn diagrams depicting the number of transcripts undergoing translation efficiency changes in cells under RS, OIS and oxidative stress (H_2_O_2_). n.s., non‐significant. Error bars indicate SEM.

In order to gain insights into translation deficiency in RS, we elaborated on identifying major deregulation patterns (Figure S3A). RS W138 fibroblasts were compared to control fibroblasts (Sabath et al., 2020). Interestingly, although ribosome stalling was observed in both control and RS cells, the overall differences in either one of the E‐, P‐ or A‐ ribosome sites and codon usage were insignificant (*p* > 0.05) (Figure 2a,b and Table S2), implying that ribosome stalling frequency may not affect translation efficiency in RS. To verify this finding, we derived Cumulative Distribution Function (CDF) curves for translation efficiency changes between the identified stalled sites in control versus RS cells, and found no statistical difference between the two groups (Figure 2b). In line with the above in vitro data, comparing cells from aged mouse kidney (Figure 2c,d and Table S2) and liver (Figure 2e,f and Table S2) to respective cells from young mice confirmed the absence of significant differences in overall ribosome stalling. As no specific uORF/dORF motifs and IRES elements were confidently called in RS cell lines or aged mouse tissue versus control, we conducted a pathway enrichment analysis including only the identified stalled genes in mouse kidney and liver (Figure S3B,C). As expected upon RS (Gorgoulis et al., 2019), pathways involved in oxidative stress were upregulated in old mice (Figure S3B), whereas biological processes such as amino acid metabolism were downregulated, indicating a state of translation deficiency (Figure S3B,C). Taken together, our results demonstrate that although translation deficiency occurs upon RS, this deficiency is likely due to a natural deterioration of the translation machinery per se and not because of distinct translation deregulation patterns.

Besides RS which may occur naturally in aging cells, extrinsic stimuli such as oxidative stress or oncogenic insults are well‐established activators of cellular senescence (Macip et al., 2002; Serrano et al., 1997; Wiley et al., 2016). By comparing RS to SIS cells (Loayza‐Puch et al., 2013; Rendleman et al., 2018; Sabath et al., 2020; Teo et al., 2019), we found significant differences in the number of genes with altered translation efficiency (Figure 2g). Specifically, RS cells displayed the lowest number of genes with translation efficiency changes, followed by SIS cells and by OIS cells, which displayed the highest number of genes undergoing translation efficiency changes (Figure 2g). Those pronounced differences compared to RS urged us to explore whether translation deregulation patterns may differ in the SIS and OIS contexts.

### 2.2 Translation deregulation upon oxidative stress may rely on uORFs

**FIGURE 3:**
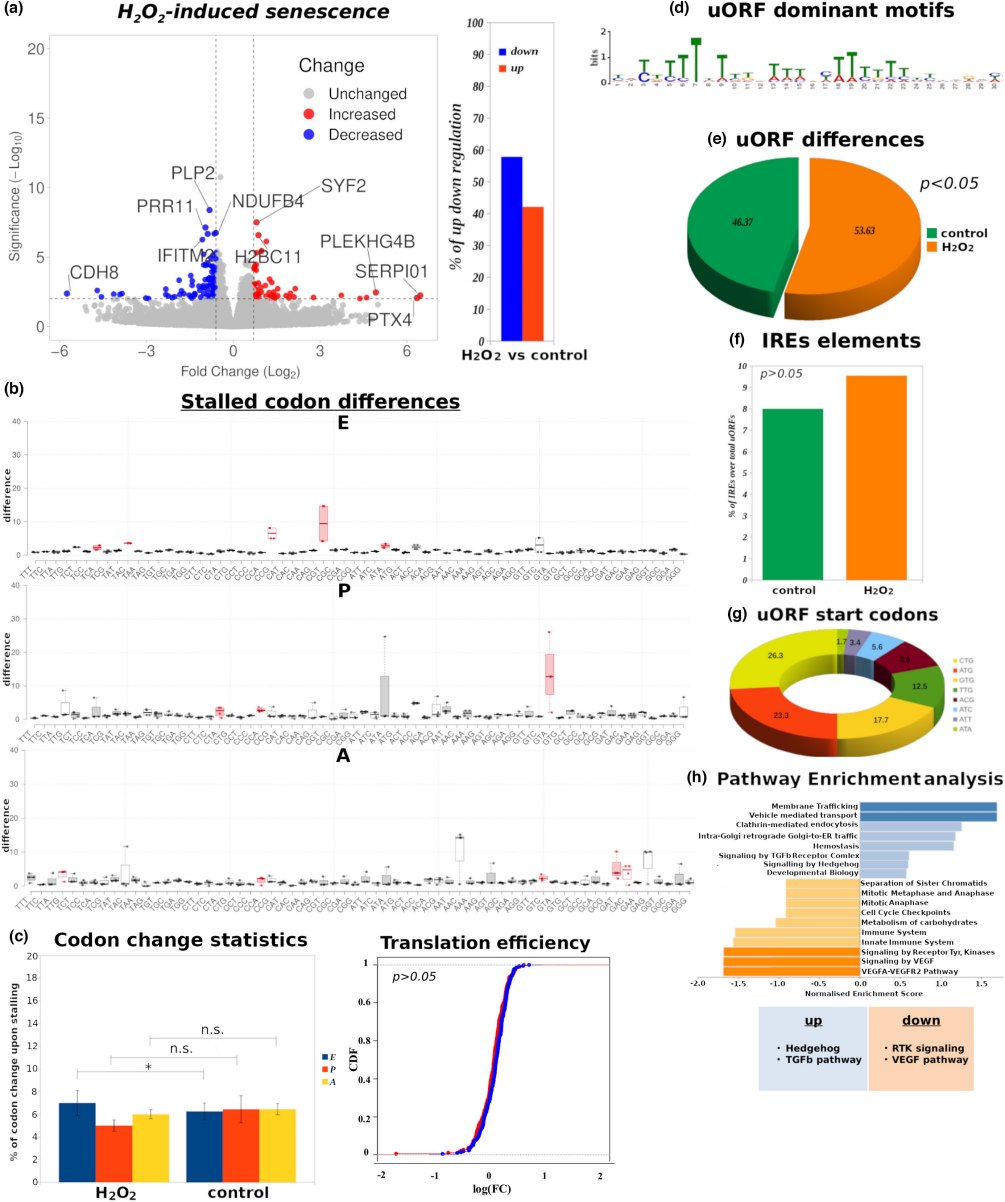
uORF‐mediated translation deregulation upon oxidative stress‐induced senescence. (a) Volcano plots demonstrating genes with significantly decreased (blue) and increased (red) translation rate in H_2_O_2_‐treated HeLa cells versus control. Bar graphs indicate the percentage of significant (*p* < 0.05) changes in translation efficiency. See also Table S2. (b) Ribosome stalling differences derived by comparing the normalized EPA coverage per codon for H_2_O_2_‐treated HeLa cells versus control. Red coloring in the box plots indicates codons where ribosomes were most stalled. (c) Left: Bar graphs indicate overall differences in the percentage of stalled codons per library in E‐, P‐ and A‐ ribosome sites between H_2_O_2_‐treated HeLa cells and control. Right: CDF curve showing an overall non‐significant difference in translation efficiency between stalled codons in H_2_O_2_‐treated HeLa cells versus untreated counterparts. (d) Identification of uORF dominant motifs using the MEME motif finding platform. CUCUU sequences resembling candidate 5′ TOP motifs are found in the identified motifs. (e) Pie chart displaying the percentage (%) of total identified uORFs found in the indicated conditions. A statistically significant increase in mRNAs carrying uORFs was observed upon H_2_O_2_ treatment versus control. (f) Bar graph displaying the percentage (%) of total IRES elements found in the indicated conditions. (g) Pie chart displaying the distribution of uORF start codons derived by observing the nt sequences at the start of the periodicity at each 5′ UTR. No differences in uORF start codons were observed between H_2_O_2_‐treated HeLa and control cells. (h). Pathway enrichment analysis for genes regulated by uORFs using the WebGestalt platform. *p < 0.05, of Student's *t*‐test; n.s., non‐significant; FDR, False Discovery Rate. Error bars indicate SEM.

Oxidative stress is one of the major causes of SIS (Macip et al., 2002; Wiley et al., 2016). We re‐analyzed published RNA‐seq and Ribo‐seq data generated from a cervical cancer cell line (HeLa) treated with hydrogen peroxide (H_2_O_2_) versus control (Rendleman et al., 2018). After 4 h of H_2_O_2_ treatment, HeLa cells displayed elevated levels of Reactive Oxygen Species (ROS) accompanied by activation of the DNA damage response, a route known to directly lead to cellular senescence in HeLa cells and other cell lines (Chen et al., 2007; Hubackova et al., 2016; Rendleman et al., 2018). Indeed, upregulation of senescence markers such as β‐Gal was evident in the examined dataset (Rendleman et al., 2018). As expected, and in line with previous results, we found that translation efficiency was decreased in HeLa cells undergoing oxidative stress versus untreated counterparts (Figures 2g and 3a; Table S2).

Differential ribosome stalling was identified when comparing H_2_O_2_‐treated HeLa cells for 4 h versus 0 h (untreated cells), in codons encoding amino acids such as proline (CCG, CCC, CCU, and CCA) (Figure 3b and Table S2). Stalling on proline codons was observed on all three ribosome sites, while the previous or next codon again encoded proline (Figure 3b). This is in line with the recent observation that diproline (Pro‐Pro) motifs may be responsible for paused ribosomes (Krafczyk et al., 2021). Overall differences in ribosome stalling between H_2_O_2_‐treated HeLa cells and controls were found to be insignificant (*p* > 0.05; Figure 3c, left). This was also reflected in CDF curves derived from stalled genes (Figure 3c, right), indicating that ribosome stalling frequency is unlikely to constitute a major translation deficiency mechanism upon oxidative stress.

Upon stress, it has been shown that ribosome coverage of the 5′ UTR of mRNAs may result in altered translation of the canonical ORF (Calvo et al., 2009). Interestingly, and in contrast to RS, our uORF/dORF identification analysis revealed the presence of dominant uORF motifs in H_2_O_2_‐treated HeLa cells, corresponding to a significant number of enriched uORFs (*p* < 0.05; Figure 3d,e and Table S2). Of note, CUCUU sequences resembling candidate 5′ TOP motifs (Avni et al., 1994; Cockman et al., 2020) were identified within uORF motifs of H_2_O_2_‐treated cells (Figure 3D). Moreover, we employed various tools such as IRESpy (Wang & Gribskov, 2019) and QGRS Mapper (Kikin et al., 2006) (see also Section 4) to identify potential IRES elements, which may promote cap‐independent translation upon cellular stress, but found no significant differences between H_2_O_2_‐treated HeLa and control cells (Figure 3f and Table S2). Based on the 3‐nt periodicity from the ribosome footprint coverage across the 3 frames on the 5′ UTR we were able to determine ATG and non‐ATG start codons in uORFs, however that start codon switch was similar in both conditions (Figure 3g). Those results indicate that translation deficiency upon oxidative stress is likely not affected by ribosome stalling or dORFs, but may be dependent on the increased number of genes controlled by uORFs.

Given that only uORFs were found enriched upon oxidative stress, we performed a uORF gene set enrichment analysis (GSEA) using the WebGestalt platform (Liao et al., 2019) to identify pathways selectively regulated by the enriched uORFs (Figure 3h). Interestingly, as it has been extensively reported upon oxidative stress‐induced senescence (OSIS) (Feng et al., 2021; Grunewald et al., 2021; Hasan et al., 2011; Tominaga & Suzuki, 2019), Hedgehog and TGF‐β signaling pathways were among the most highly upregulated pathways, whereas the VEGF pathway was ranked among the most downregulated pathways compared to control (Figure 3h). In support of this, TGF‐β/Hedgehog signaling activation and VEGF attenuation are linked with decreased mTOR activity (Dodd et al., 2015; Rosengren et al., 2018; Wu et al., 2018), which has been shown to confer a global repression of the translation machinery (Loayza‐Puch et al., 2013; Xu et al., 2014).

### 2.3 Oncogene‐induced senescence exhibits high frequency of ribosome stalling and uORF/dORF translation deregulation patterns

**FIGURE 4:**
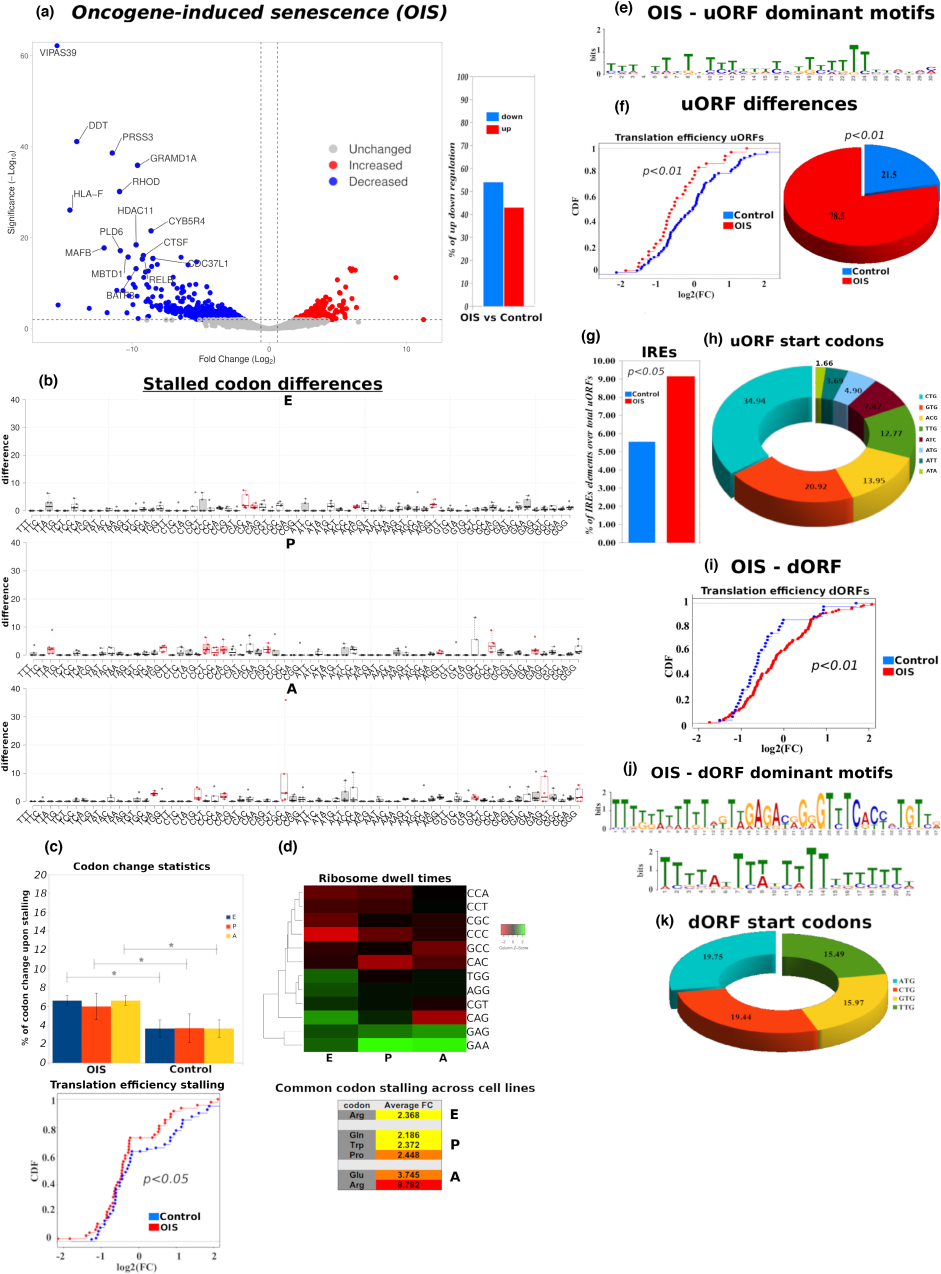
Oncogene‐induced senescence displays increased ribosome stalling, uORF/dORF patterns and IRES elements. (a) Volcano plots of genes with significantly decreased (blue) and increased (red) translation rate in human primary BJ fibroblast cells undergoing OIS versus control. Bar graphs indicate the percentage of significant (*p* < 0.05) changes in translation efficiency. See also Table S2. (b) Ribosome stalling differences derived by comparing the normalized EPA coverage per codon for OIS BJ fibroblast cells versus control. Red coloring in the box plots indicates codons where ribosomes are most stalled. (c) Top: Bar graphs indicating significant differences in the percentage of stalled codons per library in E‐, P‐ and A‐ ribosome sites between OIS and control BJ fibroblasts. Bottom: The CDF plot of the transcripts where stalling is observed shows a significant drop in translation efficiency in OIS. (d) Top: Ribosome dwell times heatmap referring to codons of the EPA ribosome sites where increased stalling may occur based on known lower translation elongation rates (Gobet et al., 2020). Combinations of codons with increased dwell times is likely to result in considerable ribosome stalling. Green color stands for increased, while red color for decreased dwell times. Bottom: Identified codon stalling resulting in respective changes in translated amino acids. (e) Identification of uORF dominant motifs using the MEME motif finding platform. CUCUU sequences resembling candidate 5′ TOP motifs are found in the identified motifs. (f) Left: The CDF curve is derived only from uORF‐carrying transcripts and exhibits a significant decrease in OIS translation efficiency versus control. Right: The pie chart shows the percentage (%) of total identified uORFs found in the indicated conditions. See also Figure S4A. (g) Bar graph with the percentage (%) of IRES elements found in uORFs of OIS versus control samples after folding the identified uORF domains with the Vienna algorithm (See also Section 4). (h) Pie chart showing the distribution of uORF start codons, with no significant differences between OIS and control BJ fibroblasts. (i) CDF plots with dORF‐carrying transcripts show significant increase of translation efficiency in OIS versus control BJ fibroblasts. (j) As in (e), dominant dORF motifs are evaluated using the MEME suite. (k) Pie chart presenting the dORF start codon distribution, with no significant changes between OIS and control BJ fibroblasts. Statistics for the CDF plots are extracted with a Wilcoxon rank sum test. *p < 0.05, of Student's *t*‐test; Error bars indicate SEM.

To investigate how OIS may impact ribosome dynamics, we re‐analyzed published RNA‐seq and Ribo‐seq datasets from immortalized human primary BJ fibroblast cells where senescence was induced by activation of the oncogenic *RAS* ^*G12V*^ gene versus control (Loayza‐Puch et al., 2013). As expected, OIS was accompanied by translation deficiency compared to normal proliferating cells (Figure 4a).

To test if ribosome stalling may contribute to translation deregulation in OIS cells, we analyzed codon occupancy by the EPA ribosomal sites (Figure 4b and Table S2). Intriguingly, we found increased stalling rates pertaining to all three ribosome sites upon OIS (Figure 4b), and subsequently identified the extent at which those codon usage differences affect production of the respective amino acids (Figure 4c,d). Of note, several Pro‐Pro motifs were again identified predominantly in the P‐ and A‐ sites. A statistical analysis of stalled codons between OIS and control confirmed the increased stalling frequency on all ribosome sites (Figure 4c, top), while a CDF curve with stalled genes demonstrated a significant drop in OIS translation efficiency in comparison to control (*p* < 0.05) (Figure 4c, bottom). Moreover, we correlated the differentially stalled codons with ribosome dwell times (Figure 4d, top), thus highlighting the codon combinations in the EPA sites (Figure 4d, bottom) which are likely to result in lower translation elongation rates upon ribosome stalling (Gobet et al., 2020).

We next sought to determine whether translation deregulation in OIS may additionally rely on potential uORF patterns. As upon oxidative stress, dominant uORF motifs were found in OIS cells; however, OIS was accompanied by a considerably higher level of uORF‐regulated genes (*p* < 0.01) (Figure 4e,f and Table S2). In support of this, a CDF curve derived only from the identified uORF‐containing transcripts exhibited a significant reduction in translation efficiency (Figure 4f). Interestingly, similarly to OSIS, CUCUU sequences were again identified within uORF motifs of OIS BJ fibroblasts, while respective uORF motifs of proliferative controls were G‐enriched (Figure 4e and Figure S4A). We next folded the RNA of the identified uORFs with the Vienna algorithm using SHAPE and DMS footprinting and estimated the presence of IRES elements (see also Section 4 for additional computational tools for IRES prediction). Interestingly, IRES elements were significantly enriched in OIS uORFs (*p* < 0.05) (Figure 4g). Upon examining the uORF start codons we observed the presence of several non‐ATG start sites, which together with IRES elements may form a potential basis for cap‐independent translation (Spriggs et al., 2008). However, there were no significant differences in alternative start codon choice between OIS and control (Figure 4h).

We then set out to assess the potential role of dORFs in OIS. Several dORFs with distinct motifs were identified mainly in OIS cells, where P‐site ribosome coverage was enriched several nt downstream the stop codon of the canonical ORF (Figure 4i,j and Table S2). Intriguingly, a CDF plot derived from dORF‐containing genes demonstrated a significant upregulation of translation efficiency in OIS compared to control (*p* < 0.01) (Figure 4i), which is in accordance with previous evidence supporting a stimulatory role of dORFs in translation regulation (Wu et al., 2020). Regarding the distribution of start codons, as with uORFs, we again found several non‐ATG start codons in dORFs (Figure 4k).

In order to understand how the presence of ribosome stalling, uORFs and dORFs may rewire cellular signaling, we pooled all transcripts of the OIS datasets carrying the above translation deregulation patterns and performed a gene prioritization analysis versus control (Figure S4B–C and Table S2). Interestingly, one of the clearly upregulated pathways was Notch, where several direct and indirect activators (TLE4, APH1A, NCSTN, APH1B and PSEN1) were found to be translated at a higher level (Figure S4D). This finding is in alignment with the established role of the Notch pathway as a mediator of cellular senescence (Teo et al., 2019).

Taken together, we provide evidence that OIS, which exhibits the highest level of gene expression alterations compared to RS or OSIS, additionally displays the highest frequency of all major translation deregulation mechanisms.

### 2.4 Human fibroblast model systems recapitulate major translation defects upon induction of senescence in vitro

**FIGURE 5:**
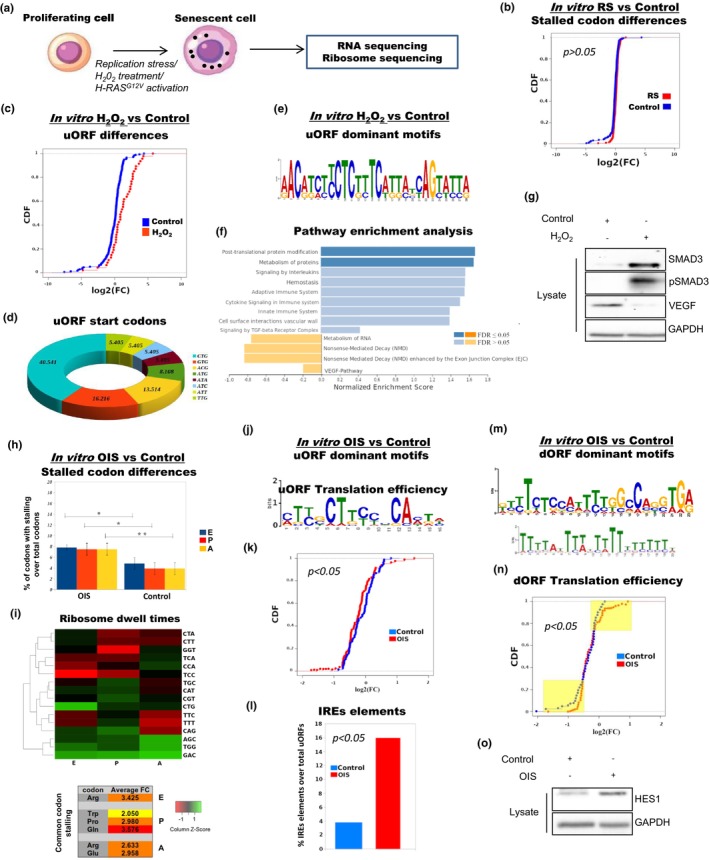
Translation deregulation patterns are experimentally recapitulated in senescence‐induced fibroblasts in vitro. (a) Schematic illustrating the experimental strategy. IMR‐90 human lung fibroblasts were forced to senesce via replication stress, H_2_O_2_ treatment or *RAS* ^*G12V*^ gene induction. Cells were subsequently lysed and subjected to RNA‐seq and ribosome profiling. See Table S2. (b) CDF curve showing non‐significant differences in translation efficiency between stalled codons in RS versus control (proliferating) IMR‐90 cells. RNA‐seq and Ribo‐seq data were retrieved by our published dataset (GEO accession number: GSE171780) (Sofiadis et al., 2021). See also Figures S5A,D,E and S6. (c) IMR‐90 cells were induced to senesce by H_2_O_2_ and subsequently compared to proliferating counterparts. The CDF curve displays an increased impact of uORFs on translation deregulation upon H_2_O_2_ treatment. (d) Pie chart displaying the distribution of uORF start codons derived by observing the nt sequences at the start of the periodicity at each 5′ UTR. (e) Identified dominant uORF motifs using the MEME platform. CUCUU motifs are again observed. (f) Pathway enrichment analysis for genes regulated by uORFs using the WebGestalt platform. See also Figures S5B,D,E and S7. (g) Western blotting of control and H_2_O_2_‐treated cell lysates with indicated antibodies, verifying enhancement of TGF‐β signaling (SMAD3, pSMAD3) and downregulation of VEGF signaling upon H_2_O_2_‐induced senescence. These observations are in line with our published dataset analyses. See also Figure S7F. (h) Bar graphs indicating significant differences in the percentage of stalled codons per library in E‐, P‐ and A‐ ribosome sites between OIS and control IMR‐90 fibroblasts. (i) Ribosome dwell times heatmap and common codon stalling in E‐, P‐ and A‐ sites. Green color stands for increased, while red color for decreased dwell times. Those in vitro results are in agreement with our published OIS versus control dataset analyses. See also Figures S5C–E and S8A–C. (j) Identification of uORF dominant motifs using the MEME platform in OIS versus control IMR‐90 cells. CUCUU sequences were detected (potential TOP‐like motifs). (k) The CDF curve is derived only from uORF‐carrying transcripts and exhibits a significant decrease in OIS translation efficiency versus control. See also Figures S7D and S8D. (l) Bar graph demonstrating an increased percentage (%) of IRES elements in uORFs of OIS versus control cells. (m) Dominant dORF motifs were similar to the ones identified in our published dataset analyses. See also Figure S8E. (n) CDF plots with dORF‐carrying transcripts show significant increase of translation efficiency in OIS versus control IMR‐90 fibroblasts. (o) Western blotting in control (proliferating) and OIS IMR‐90 cell lysates with indicated antibodies, verifying enhancement of Notch signaling via HES1 upregulation. See also Figure S8F,G. Statistics for the CDF plots are extracted with a Wilcoxon rank sum test. FDR, False Discovery Rate. Vectors were obtained from www.vecteezy.com.

Ribosome footprints are often sensitive to biases resulting from protocol differences and library preparations (Hussmann et al., 2015; Tunney et al., 2018). Therefore, to experimentally challenge the validity of the above observations, we implemented IMR‐90 human lung fibroblasts, which we led to senescence through replicative exhaustion, H_2_O_2_ treatment or inducible *RAS* ^*G12V*^ activation (Figure 5a), thus recapitulating RS, OSIS and OIS, respectively. After validating successful senescence induction versus controls according to the established senescence detection multimarker algorithm (Kohli et al., 2021) (Figure S5A–E), cells were subjected to RNA‐seq and Ribo‐seq (Figure 5a and Table S2).

In keeping with our previous analyses on published RS datasets, we found that RS IMR‐90 cells displayed increased translation deficiency compared to controls (Figure S6A and Table S2). Moreover, no significant differences were generally detected in codon occupancy by EPA ribosome sites in RS versus control cells (Figure S6B), which was further verified by CDF curves showing insignificant translation efficiency changes (*p* > 0.05) between identified stalled sites (Figure 5b). Given that we failed to identify other potentially prevalent translation deregulation mechanisms, our experimental results corroborate the finding that RS may not be accompanied by distinct translation deregulation patterns.

Along the same lines, we found reduced translation efficiency between our H_2_0_2_‐treated IMR‐90 cells and untreated counterparts (Figure S7A and Table S2); however, this was not accompanied by significant differences in ribosome stalling (Figure S7B,C). In accordance with our uORF/dORF analysis on already published datasets, uORFs were found enriched in our H_2_O_2_‐induced IMR‐90 cells (Figure 5c), while several uORF start codons other than ATG were consistently identified, with CTG being again the most dominant (Figure 5d). CUCUU sequences comprising potential 5′ TOP motifs (Avni et al., 1994; Cockman et al., 2020) were again detected within those identified uORF motifs, in contrast to G‐enriched repeats found in uORF motifs of proliferative IMR‐90 controls (Figure 5e and Figure S7D). Consistent with published datasets, our analysis showed non‐significant differences in the presence of predicted IRES elements between OSIS IMR‐90 cells and proliferative controls (Figure S7E and Table S2). Moreover, we conducted a pathway enrichment analysis on uORF‐regulated genes, and importantly, the TGF‐β and VEGF pathways emerged again among the most highly upregulated and downregulated pathways, respectively, compared to control (Figure 5f). We experimentally confirmed those correlations using SMAD3 protein expression and phosphorylation, as well as total VEGF protein levels as readouts for TGF‐β and VEGF pathway activation, respectively (Figure 5g and Figure S7F). These results provide confirmation that translation deficiency upon oxidative stress may be primarily dependent on uORF‐mediated gene regulation, resulting in selective modulation of signaling pathways known to be involved in OSIS (Grunewald et al., 2021; Hasan et al., 2011; Tominaga & Suzuki, 2019).

We next utilized IMR‐90^ER:RAS^ cells which were forcibly led into OIS via 4‐hydroxy‐tamoxifen (4‐OHT)‐mediated induction of *RAS* ^*G12V*^ expression. As expected, OIS IMR‐90 cells displayed impaired translation efficiency compared to proliferating IMR‐90 cells (Figure S8A and Table S2). Our experimental analysis demonstrated significantly higher levels of ribosome stalling in EPA sites of OIS versus control cells, while the translation efficiency of stalled genes was significantly lower in OIS (*p* < 0.05; Figure 5h, Figure S8B,C). Ribosome dwell times in OIS IMR‐90 cells yielded similar codon stalling patterns to respective published datasets (Figure 5i). For example, in both experimental and published datasets, arginine (Arg) coding codons were identified as predominant stalling codons of the E‐ and A‐ ribosome sites, while similar codons were also identified in the P‐ ribosome site (Figure 5i, bottom and Figure 4d, bottom). Our experimental data additionally confirmed that OIS cells were significantly enriched in uORFs (containing again CUCUU repeats) compared to controls, which led to impaired translation efficiency (*p* < 0.05; Figure 5j,k), accompanied by additional enrichment of IRES elements (*p* < 0.05; Figure 5l). Dominant dORF motifs were again identified only in OIS IMR‐90 cells, while CDF curves derived from dORF‐containing genes displayed a marked increase in translation efficiency (*p* < 0.05) (Figure 5m,n). Notably, uORF and dORF start codon choice in OIS IMR‐90 cells was highly similar to that of published OIS datasets, with CTG being the predominant uORF and ATG the predominant dORF start codon (Figure S8D,E). Lastly, a pathway enrichment analysis in OIS IMR‐90 cells versus control cells highlighted again Notch as one of the most upregulated pathways (Figure S8F). To experimentally confirm this finding, we assessed the protein levels of the terminal Notch pathway effector HES1 (Chen et al., 1997; Ishibashi et al., 1995) in OIS IMR‐90 cells compared to proliferating counterparts, and indeed, found an increased expression of HES1 upon OIS (Figure 5o and Figure S8G).

Our experimental findings recapitulate the observations made from published in vitro and in vivo human and mouse datasets, thus strongly indicating that different senescence stimuli are accompanied by defined sets of translation deregulation mechanisms engaging distinct signaling pathways. OIS may accumulate the highest level of deregulation patterns compared to other common forms of SIS such as oxidative stress. On the contrary, RS is free of such patterns, indicating that manifestation of translation deregulation mechanisms/patterns may be directly proportional to stress severity.

## 3 DISCUSSION

**FIGURE 6:**
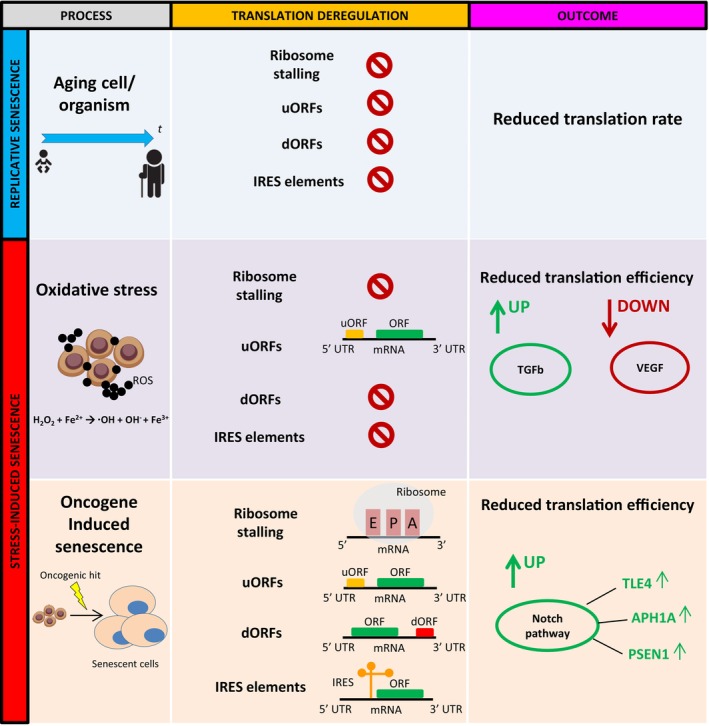
Model. Although the frequency of translation deregulation mechanisms is similar between aged cells undergoing RS and young counterparts, SIS displays a clear manifestation of deregulation patterns. In the case of OSIS, uORFs are significantly enriched compared to control, which correlates with upregulation/downregulation of distinct signaling pathways. OIS, on the contrary, is characterized by significant enrichment of ribosome stalling, uORF/dORF patterns and IRES elements, accompanied by upregulation of Notch signaling activators. Our model demonstrates that the differential rates of translation deregulating mechanisms may be hallmarks of separate types of cellular senescence. Vectors were obtained from www.vecteezy.com.

In this study, we re‐analyzed published RNA‐seq and Ribo‐seq datasets, in which the translation defects seen upon cellular stress or senescence were initially examined only from a gene expression or proteostasis perspective. By implementing various computational approaches, we addressed the so far unanswered question of whether distinct translation deregulation mechanisms may occur upon various types of senescence. Our data demonstrate that the frequency of mechanisms such as ribosome stalling in aged cells undergoing RS was no different from controls, whereas those mechanisms were significantly more frequent in SIS (Figure 6). Interestingly, while only uORF‐mediated deregulation is likely to accompany SIS, all translation deregulation patterns (ribosome stalling, uORFs/dORFs and IRES elements) are present in cells undergoing OIS, potentially modulating distinct signaling pathways (Figure 6). Thus, RS, SIS and OIS may be distinguished based on the incremental accumulation of translation deregulation mechanisms, which is completely aligned with their translation deficiency status. Importantly, those observations were experimentally confirmed in human fibroblast model systems, hence augmenting the validity of the findings.

Our analyses of in vivo datasets verify that translation deficiencies are progressively less marked when old mice are compared to gradually older mice. Translation deficiency has already been identified as an accompanying trait of cellular senescence (Lessard et al., 2018; Nishimura et al., 2015) and the old mice that we implement in our “old vs. young” comparisons (26‐ and 32‐month‐old mice) display increased senescence markers (Anisimova et al., 2020). While we cannot exclude the possibility that other factors may contribute to translation deficiency, it is known that senescence is indeed established early in life (even during embryonic development) and the number of senescent cells increases with age in several tissues (Dimri et al., 1995; Krishnamurthy et al., 2004; Liu et al., 2009; Melk et al., 2004).

In all our examined datasets (RS, OSIS and OIS versus respective controls), either published or experimental, we found that of the genes whose expression is altered, the percentage of downregulated genes was always found higher than that of upregulated genes, pointing to an overall loss of gene expression. However, a portion of genes were found upregulated in each case. As senescence has been also associated with high translational and metabolic activity reflected in SASP (Dorr et al., 2013; Herranz et al., 2015; Laberge et al., 2015; Narita et al., 2011), we conducted a pathway enrichment analysis to unveil the identity of upregulated genes (Figure S2A), and found a large portion of stress‐related genes, again in accordance with the expected senescence phenotype (Rivera‐Mulia et al., 2018). Moreover, it is entirely possible that genes with elevated expression may be linked to pathways shown to contribute to the senescence phenotype (Pantazi et al., 2019).

Of note, an interesting point emerging from our published dataset analyses was the similar percentages of under‐translated transcripts in all senescence types (around 55%–60%, Figures 1a, 3a and 4a). The above percentages, however, should be interpreted together with the number of identified transcripts with translation changes, which were 161, 307 and 703 in RS, SIS and OIS, respectively (Figure 2g). It is, therefore, clear that OIS displays the highest level of translation deficiency, whereas RS the lowest. A potential explanation regarding the higher degree of translation deficiency found in OIS compared to OSIS may derive from the fact that oxidative stress itself constitutes an early event in oncogenic transformation, often stimulating tumorigenic growth (Hayes et al., 2020).

Elements of translation regulation such as sites of ribosome stalling/frameshifting, uORFs and dORFs may act as regulatory switches of translation upon stress toward maintaining cell functionality. Furthermore, the prediction of IRES elements together with the identification of non‐canonical translation sites (non‐ATG) provides further insights into alterations of RNA structure upon stress. Such structural conformations deserve to be deeply investigated genome‐wide upon stress signals, using chemical probing techniques.

The Notch signaling pathway was identified as one of the activated pathways in OIS when we analyzed the transcripts that are associated with translation deregulation mechanisms. Although signaling pathways are largely context‐dependent, Notch is considered to be a developmental pathway which is normally regulated in an oscillatory fashion (Ivanov, 2019; Shimojo et al., 2014). Perturbation of this regulated pattern is accompanied by pathological conditions, such as defective neurogenesis or tumorigenesis (Kageyama et al., 2018). In our analysis, the Notch pathway is activated together with other pathways, such as p53 (Figure S4B and Table S2). Interestingly, rapamycin‐mediated mTOR inhibition was previously found to boost Notch activity (Shepherd et al., 2013; Vo et al., 2011) and p53 was shown to also inhibit mTOR, a global translation inducer (Loayza‐Puch et al., 2013). A recent link was established between Notch signaling and the tumor suppressor RASSF1A, which functions as a scaffold of the Hippo pathway, a master developmental pathway controlling organ growth in mammals (Papaspyropoulos et al., 2022). RASSF1A is also known to regulate p53 stability (Song et al., 2008), hence it would be interesting to explore the potential involvement of the Hippo pathway in regulating translation in senescence, and particularly, in cells under OIS.

It has been recently shown that under certain conditions, a subset of cells in a senescent population resume proliferation by re‐entering the cell cycle, a phenomenon referred to as escape from oncogene‐induced senescence (EOIS) (Galanos et al., 2016; Patel et al., 2016). We recently identified a chromosomal inversion leading to activation of the circadian transcription factor *BHLHE40* as a driver of EOIS (Zampetidis et al., 2021). Given that OIS displays the highest frequency of translation deregulation mechanisms, it would be interesting to assess if those mechanisms are rewired in genes such as *BHLHE40* to re‐establish efficient translation, as the cell undergoes extensive chromatin changes to resume proliferation and acquire an aggressive phenotype.

Despite the importance of translation deregulation patterns depending on the insult, translation deficiency may not solely rely on those patterns. For example, our dataset provides clear evidence of an increased ribosome stalling frequency in OIS. However, ribosome stalling on a specific codon affecting expression of a key translation machinery gene would potentially also lead to translation deficiency. This shows that, apart from the identified mechanisms, individual gene‐dependent factors may also contribute to the severity of translation impairment. Additionally, metabolomics analyses in order to measure free amino acid availability (Bidlingmeyer et al., 1984) and analysis of charged tRNAs (Evans et al., 2017) would potentially shed light on codon overrepresentation observed upon OIS in each one of the three EPA ribosome sites.

In summary, we show that translation deficiency may occur upon cellular senescence, and that this phenotype occurs at different rates in different senescence subsets. By elaborating on major translation deregulation mechanisms/patterns (ribosome stalling, uORF/dORF patterns and IRES elements), we find that RS displays lower translation deficiency which is not accompanied by major deregulation mechanisms. Nevertheless, this is not the case in SIS, where oxidative stress displays strong uORF enrichment, and most importantly in OIS, where a significantly higher rate of all major deregulation mechanisms is clearly identified as predominant. We demonstrate that manifestation of translation deregulation mechanisms is directly proportional to the level of translation deficiency. Moreover, our pathway enrichment analyses from both published and in vitro datasets consistently identified TGF‐β/VEGF and Notch pathways as potential mediators of translation deficiency in OSIS and OIS, respectively.

## 4 METHODS

### 4.1 Experimental analysis

#### 4.1.1 Cell lines and reagents

Human lung fibroblasts (IMR‐90, ATCC, passage number: 5) and Phoenix™‐Ampho (ATCC) cells were maintained in Dulbecco's High‐Glucose Modified Eagle Medium (DMEM), containing 10% fetal bovine serum and 1% Penicillin–Streptomycin, and incubated at 37°C with 5% O_2_ and 5% CO_2_.

#### 4.1.2 Plasmids, retrovirus production and RAS transduction

Phoenix cells were seeded in T75 flasks 1 day prior to transfection, and were subsequently transfected with 1 mL of transfection mix in 6 mL of complete DMEM for 48 h. The transfection mix was prepared with 1 μg of pLNCX2 ER:ras (Addgene #67844) per 100 μL of Opti‐MEM™ (ThermoFisher Scientific) and 2% TurboFect™ (ThermoFisher Scientific). The retrovirus‐containing medium was collected, filtered, and then used to transduce IMR‐90 cells. After 24 h of transduction, IMR‐90 cells were incubated with a selection medium comprised of complete DMEM + G‐418 (400 μg/mL). After 10 days of selection, IMR‐90^ER:RAS^ cells were maintained in complete DMEM + G‐418 (200 μg/mL).

#### 4.1.3 Senescence induction

For replicative senescence, IMR‐90 cells were continuously passaged to replicative exhaustion as previously described (Sofiadis et al., 2021). For H_2_O_2_‐induced senescence, IMR‐90 cells were treated with 600 μM H_2_O_2_ twice, as also described previously (Chen et al., 2007). For oncogene‐induced senescence, RAS was activated by treating the IMR‐90^ER:RAS^ cells with 100 nM of 4‐hydroxy‐tamoxifen (4‐OHT) for 7 days. Senescence was verified 7 days post induction.

#### 4.1.4 RT‐qPCR analysis

PureLink™ RNA Mini Kit (ThermoFisher Scientific) was used to lyse cells and extract total RNA. The concentration and purity of the RNA were measured using NanoDrop™. cDNA was generated using the High‐Capacity cDNA Reverse Transcription Kit (ThermoFisher Scientific), and subsequently amplified using GoTaq® DNA Polymerase (Promega) in a LightCycler 480 Instrument II (Roche). The following primers were used: *p16* ^*INK4A*^ FW: CCCAACGCACCGAATAGTTA and REV: ACCAGCGTGTCCAGGAAG, *p21* ^*WAF1/Cip1*^ FW: ACTGTCTTGTACCCTTGTGCC and REV: CGGCGTTTGGAGTGGTAGAA, *LMNB1* FW: GTGCTGCGAGCAGGAGAC and REV: CCATTAAGATCAGATTCCTTCTTAGC and *TUBA1A* FW: CTTCGTCTCCGCCATCAG and REV: CGTGTTCCAGGCAGTAGAGC. Relative gene expression analysis was done using the Livak method ($2^{-\Delta \Delta C_{t}}$) (Livak & Schmittgen, 2001). Tubulin was used as a reference gene for the normalization of *C*_t_ values.

#### 4.1.5 Immunocytochemistry

IMR90 fibroblasts were seeded on coverslips, fixed using 4% PFA diluted in PBS for 10 min at 4°C, and permeabilized by applying Triton‐X 0.3% in PBS for 15 min. Cells were washed with 1× PBS and incubated with 3% H_2_0_2_ for 18 min at RT in order to block endogenous peroxidase activity. The coverslips were washed with 1× PBS and incubated in goat serum for 1 h in RT (Abcam; ab138478) serving as blocking solution. Cells were subsequently incubated with primary anti‐Ki67 antibody (1/200, Abcam; ab16667) for 1 h at RT. Development of positive signal was carried out using the Dako REAL EnVision Detection System (K5007), according to the manufacturer's instructions. Cells were counterstained with hematoxylin, mounted and observed on a Zeiss Axiolab 5 microscope using the 20× objective.

### 4.2 SenTraGor™ staining for senescence detection

For SenTraGor™ staining, cells seeded on coverslips were treated with 50% and then 70% ethanol, for 5 min each. SenTraGor™ was applied for 10 min at 37°C. Coverslips were washed with 50% ethanol for 2 min and then washed again with 1X PBS. Potentially remaining amounts of SenTraGor™ were removed by washing with 0.3% Triton‐X diluted in PBS, for 3 min. Cells were washed with 1X PBS and an anti‐biotin primary antibody (dilution 1:300, Abcam; ab201341) was applied for 1 h at RT. Development of positive signal and microscopy was performed as described in the Section 4.1.5.

### 4.3 RNA‐sequencing

Cells were harvested 14 days post senescence induction. Growth media was removed, and the cells were washed with cold PBS containing 100 μg/mL of cycloheximide. Cells were subsequently scraped and pelleted, and later stored at −80°C until they were dispatched for sequencing. Stranded mRNA‐seq libraries were generated from flash frozen cell pellets. Cell pellets were lysed in ice‐cold polysome lysis buffer (20 mM Tris pH 7.5, 150 mM NaCl, 5 mM MgCl_2_,1 mM DTT, 1% Triton X‐100) supplemented with cycloheximide (100 μg/mL). For stranded mRNA‐seq, total RNA was extracted from 10% of lysate using TRIzol, before mRNA was poly(A)‐enriched, fractionated, and converted into Illumina compatible cDNA libraries. Stranded mRNA‐seq libraries were sequenced 150PE on Illumina's Nova‐seq 6000 platform to depths of 20 million raw read pairs per sample.

### 4.4 Ribo‐sequencing

For Ribo‐seq, the remaining lysates which were not used for RNA‐seq were RNase‐ treated before ribosomes were enriched by size exclusion chromatography using MicroSpin S‐400 HR columns. Following RNA purification and size selection of ribosome protected mRNA fragments on 15% urea PAGE gels, contaminating rRNA was depleted from samples using EIRNA Bio's custom biotinylated rRNA depletion oligos before the enriched fragments were converted into Illumina compatible cDNA libraries. Ribo‐seq libraries were sequenced 150PE on Illumina's Nova‐seq 6000 platform to a depth of 100 million raw read pairs per sample.

### 4.5 Immunoblotting

Immunoblotting from cell lysates was performed as previously described (Papaspyropoulos et al., 2022). The following primary antibodies were used at a concentration of 1:1000; HES1 (AB5702), GAPDH (Cell Signaling #5174), SMAD3 (ThermoFisher #51–1500), pSMAD3 (Cell Signaling #9520), VEGF (Santa Cruz; sc‐507) and HRP‐linked anti‐rabbit secondary antibodies were used at a concentration of 1:1000 (Cell Signaling #7074).

### 4.6 Computational analysis

#### 4.6.1 Quality filtering and alignments

FASTQ files were retrieved immediately after sample sequencing for in vitro experiments or downloaded from Gene Expression Omnibus (GEO) (https://www.ncbi.nlm.nih.gov/geo/) (Table S1) for published datasets. Quality filtering for each library was performed with the FastQC tool (https://www.bioinformatics.babraham.ac.uk/projects/fastqc/). To detect over‐represented adapter sequences, the small utility program Minion was used (https://www.ebi.ac.uk/research/enright/software/kraken) (Davis et al., 2013). Adapter trimming was accomplished with Cutadapt (Martin, 2011), where the quality trimming threshold was set to 20 with a minimum read length set to 21nt. RNA‐seq samples were mapped using STAR aligner using mm10, hg19 and hg38 indexes. Ribosome profiling data were aligned with TopHat aligner (Trapnell et al., 2009) preferably using parameters bowtie1‐g1. For the downstream analysis, we examined the fragment read length frequency per library with an in‐house Perl script and also determined the 3‐nt periodicity using the scripts from the RiboTaper algorithm (Calviello et al., 2016).

When ribosome protected fragments (RPFs) were aligned to mRNA sequences, the majority of the RPF ends were located at a specific distance from the first nucleotide of the A‐site codon of the elongating ribosome. Then, the RPFs aligned predominantly to either the first or third position of the A‐site codon and the second position presented the lowest coverage of RPFs. This pattern was repeated along the CDS, thus forming a periodic signal. To determine the P‐site offset, we looked for a high start codon peak that could be seen at some distance upstream of the annotated start codon, and that distance corresponded to the offset. Via the previous method we tried to infer the appropriate read lengths and P‐site offsets to be used for downstream analysis per library (usually +12‐nt from the read start for a 28‐, 29‐ and 30‐nt ribosome footprint read fragment). Similarly, we assessed periodicity over smaller fragments of 21‐nt read length.

#### 4.6.2 Translation efficiency and mRNA abundance changes

To estimate changes in mRNA abundance regarding the RNA‐seq analysis we used featureCounts (Liao et al., 2014) from the Rsubread package to estimate the read counts per gene and the edgeR algorithm (Robinson et al., 2010) to determine RNA abundance differences. For translation efficiency changes, the Deseq2 pipeline was implemented (Love et al., 2014) after filtering the reads while excluding non‐coding RNAs, tRNAs, rRNAs, miRNAs and snoRNAs. Estimation of potential differentially spliced isoforms regarding the RNA‐seq analysis was carried out using the cufflinks platform (Trapnell et al., 2012) and DEX‐seq package (Soneson et al., 2016). Differentially translated mRNAs and differential mRNA abundance were determined based on a transcript p‐value cut‐off (<0.05), a log2FC cut‐off (≥0.65) or a log2FC cut‐off (≤−0.65).

#### 4.6.3 Codon usage frameshifts and ribosome stalling analysis

To estimate codon usage, we evaluated the P‐site read coverage based on the scripts from the RiboTaper algorithm (Calviello et al., 2016). The P‐site offset position is calculated using RiboTaper, where we evaluate the P‐site density in the 3 frames while examining ribosome fragments of various lengths. A periodic signal usually starts +12nt downstream the start of a 28‐, 29‐ or 30‐nt read fragment. The P‐site read coverage over the CDS per transcript is estimated by using the coverageBed command from the BEDTools package (Quinlan & Hall, 2010). Having the P‐sites we obtained the E‐ and A‐ sites as ±3nt from the P‐site genomic coordinates. Around the Ribosome EPA sites we obtained the FASTA sequence using the fastaFromBed command from BEDTools. Subsequently, we estimated the total read coverage per codon versus the total coverage for each codon per sample where, by combining the replicated samples, we derived statistics per codon and estimated differences between conditions (see also Section 4.7).

To estimate candidate ribosome stalling sites, we looked for sites with high ribosome occupancy per P‐site coverage of more than ~4 standard deviations of the total average P‐site coverage per sample. This is evaluated after merging the P‐site CDS coverage per transcript and estimating the overall average P‐site coverage per sample. These high density sites are frequently accompanied by a frameshift (Chang & Wen, 2021). Potential frameshifting was identified by examining a region of 120‐nt around the stalling site. A minus 1 frame‐shift will result in a change of the observed periodicity in the 3 frames in the 120‐nt window. The periodicity in the 3 frames can be visualized as the ribosome densities in the three frames (Michel et al., 2012). To that end, we also assessed if high occupancy sites can occur 60‐nt upstream and/or 60‐nt downstream of the stalled P‐site location to potentially refer to colliding ribosomes or disomes (Meydan & Guydosh, 2020). Normalization with the RNA‐seq data excluded potential artifacts and over‐sequenced fragments. If less than 10 reads were identified per transcript regarding the RNA abundance and the Ribosome coverage, then these transcripts were eliminated from the analysis.

#### 4.6.4 Detection of uORFs and dORFs

To estimate the relative abundance of uORFs or dORFs we used the Thomson multi‐tapping approach (https://cran.r‐project.org/web/packages/multitaper/multitaper.pdf) implemented by RiboTaper (Calviello et al., 2016). All P‐sites per library are first mapped relative to the 5′ UTR and 3′ UTR genomic coordinates. The signal to enter the multi‐tapping approach comes from the P‐site read occupancy on the 5′ UTR or 3′ UTR and is organized per transcript domain. A table which holds the P‐site coverage across the 5′ UTR or 3′ UTR per transcript is then tested for the 3‐nt periodicity according to the Thomson multi‐tapping frequency result, where a translated region leads to a high signal of 0.33 Hz as translation occurs in every 3 codons (Calviello et al., 2016). The initiation and start of a periodic signal is estimated and codon usage is determined. The start codons in uORFs are estimated through an area of 60nt around the start of periodicity. If ATG is found within the 60‐nt window, then uORFs are regarded as ATG starting, otherwise possible alternate start codons are examined based on the frame where best periodicity was detected. Thus, this gives us the ability to determine translation start sites alternative to ATG. Alternate isoforms where a CDS overlaps the 5′ and 3′ UTRs were excluded from the analysis. uORFs starting at a distance of more than 20‐nt from the main start of the canonical ORF CDS were only included in the analysis, in order to accurately determine a true periodicity before the canonical downstream ORF. The same analysis was performed for potential dORFs. To analyze the 3‐nt signal found in the 5′ UTR, to assure the prediction of the uORF start codons and to identify potential artifacts, we overlapped the predicted uORFs from our analysis with that of McGillivray et al. (2018) (McGillivray et al., 2018) where we find a more than 50% overlap for uORFs under normal conditions, with good coverage and score.

#### 4.6.5 Consensus motif and RNA structure enrichment

To estimate specific motif enrichment around Ribosome stalling sites and u/dORFs, the MEME suite (Bailey et al., 2015) and cERMIT software (Georgiev et al., 2010) were employed. Regarding the RNA structure, DMS‐seq and icSHAPE (Flynn et al., 2016; Rouskin et al., 2014) chemical probing methods were used to guide the RNA folding using the Vienna algorithm (Lorenz et al., 2011). To determine the consensus RNA structure motifs, the Beam software was used (Pietrosanto et al., 2018). Additionally, RNA folding structures from Vienna were converted with the RNA Lib from Vienna (https://www.tbi.univie.ac.at/RNA/ViennaRNA/doc/html/index.html), across a 150nt region to letters demonstrating whether regions along the 150nt fragments belong to a multi‐loop, internal loop or hairpin. An overrepresenting k‐mer analysis was then used to estimate the structure around the Ribosome stalling domains. To detect IREs elements, we used several tools such as IRESpy (Wang & Gribskov, 2019) or QGRS Mapper (Kikin et al., 2006). Moreover, we used the RNA forester method from Vienna (https://www.tbi.univie.ac.at/RNA/RNAforester.1.html) to extract similarity scores based on multiple alignments from structures downloaded from the Human IREs Atlas (http://cobishss0.im.nuk.edu.tw/Human_IRES_Atlas/DataStatistics) and functionally tested structures (Zhao et al., 2020).

#### 4.6.6 Gene set enrichment analysis (GSEA)

Gene set enrichment analysis was performed with the WebGestalt platform (Liao et al., 2019) by uploading the gene names and log2 fold change (log2FC) translation efficiency changes, in order to retrieve the normalized scores of enriched pathways when comparing normal versus aged or senescent stimuli. We also used the Enrichr platform (https://maayanlab.cloud/Enrichr/) (Chen et al., 2013) to evaluate the enriched pathways and gene sets. Furthermore, we used a combined score for gene sets with over 5 genes, where we integrated the p‐value threshold with a directionality consensus score. Directionality was determined by using the mean value of the log2FC of the mRNA levels of the genes forming the pathway.

#### 4.6.7 Gene prioritization analysis

The genes with identified uORFs/dORFs and ribosome stalling were matched with their translation efficiency changes. For these sets of genes we performed a network and pathway analysis to identify gene‐to‐gene interactions per pathway. Regarding the network and pathway analysis we used WebGestalt (Liao et al., 2019), Enrichr (Chen et al., 2013), esyN (Bean et al., 2014) and ConsensusPathDb (Kamburov et al., 2011). A score per gene is obtained with the following score of importance (S) formula:

$$\begin{array}{c} S=\left(-\log\left(p\;\text{value of}\;\mathrm{TE}\right)–\log\left(p\;\text{value of the significant pathway where the gene belongs}\right)\right)*\;{\text{TElog}}_{2}\mathrm{FC}*\;{\text{Connections}}_{\mathrm{per}\;\text{gene}}, \end{array}$$

where TE stands for translation efficiency, FC for fold change and

$${\text{Connections}}_{\mathrm{per}\;\text{gene}}=\frac{\text{number of gene connections}}{\text{total connections of the network}}$$

### 4.7 Statistical analysis

Differential codon usage between normal conditions versus aged or senescent stimuli were estimated with Student's *t test* between the compared conditions. In order to derive codon usage differences between compared conditions, the average normalized occupancies per codon (which is the codon occupancy divided by the total occupancy across the codons per library) for the aged or senescent conditions was divided by the average normalized occupancy obtained from the normal conditions. For example, codon occupancy for codon CCG is derived using the formula:

$${\mathrm{CCG}}_{\text{repl}1\text{senescence}}=\frac{\sum_{i}^{n}{\text{PCCG}}_{i}}{\sum_{i}^{n}P{\mathrm{ATG}}_{i}+\ldots \sum_{i}^{n}{\text{PCCG}}_{i}+\ldots \sum_{i}^{n}P{\mathrm{CCT}}_{i}+\ldots \sum_{i}^{n}P{\text{codon}}_{64}},$$

where $\sum_{i}^{n}{\text{PCCG}}_{i}$ is the total occupancy of the stalled codon CCG in the sample and *n* are the stalling positions on each displayed codon. The differential codon usage is derived using the formula:

$$\text{Differential codon usage}\;\mathrm{per}\;\text{replicate}=\frac{\left({{\mathrm{CCG}}_{\text{repl}1}}_{\text{senescence}}\right)}{\left({{\mathrm{CCG}}_{\text{repl}1}}_{\text{normal}}\right)}$$

To define the differences observed between conditions in terms of translation efficiency when examining uORFs or dORFs and stalling sites we obtained the log2FC of the transcripts where uORFs/dORFs or stalling were found and estimated the cumulative density function (CDF) of the translation efficiency. P‐values were extracted using the Wilcoxon Rank Sum Test comparing the distributions of the normal translation efficiency versus those of the senescent stimuli.

For all experiments reported in this manuscript, at least 3 biological replicates were used and statistical significance was determined by Student's *t* test.

## AUTHOR CONTRIBUTIONS

An.P. perfomed in vitro experiments and interpreted experimental and computational data, O.H. analyzed and interpreted computational data, and A.A. performed in vitro experiments and interpreted experimental data. Ai.P., P.V., K.E., M.F. and Ar.P. contributed to data interpretation. An.P., O.H., M.D. and V.G. wrote the manuscript. M.D. and V.G. supervised the study. All authors read and approved the final manuscript.

## CONFLICT OF INTEREST STATEMENT

M.D. is a co‐founder and shareholder of Cleara Biotech and an advisor for Oisin Biotechnologies. The laboratory of M.D. received funding from Cleara Biotech, Oisin Biotechnologies and Ono Pharmaceuticals.

## Supporting information


Figure S1

Figure S2

Figure S3

Figure S4

Figure S5

Figure S6

Figure S7

Figure S8

Table S1

Table S2

Appendix S1

## Data Availability

All the code scripts that were used in this study are available at the following repository links: https://github.com/Orsalia/Ribosome_analysis and https://github.com/Orsalia/scRNA‐seq‐examples. All datasets generated in this study have been deposited in the GEO database (accession number: GSE225095).
