# Supplementary figures and images for: Decoding of translation‐regulating entities reveals heterogeneous translation deficiency patterns in cellular senescence
Source: Aging Cell. 2023 Aug 7;22(9):e13893. doi: 10.1111/acel.13893 (PMC10497830; doi:10.1111/acel.13893)

# Supplementary Figure 1

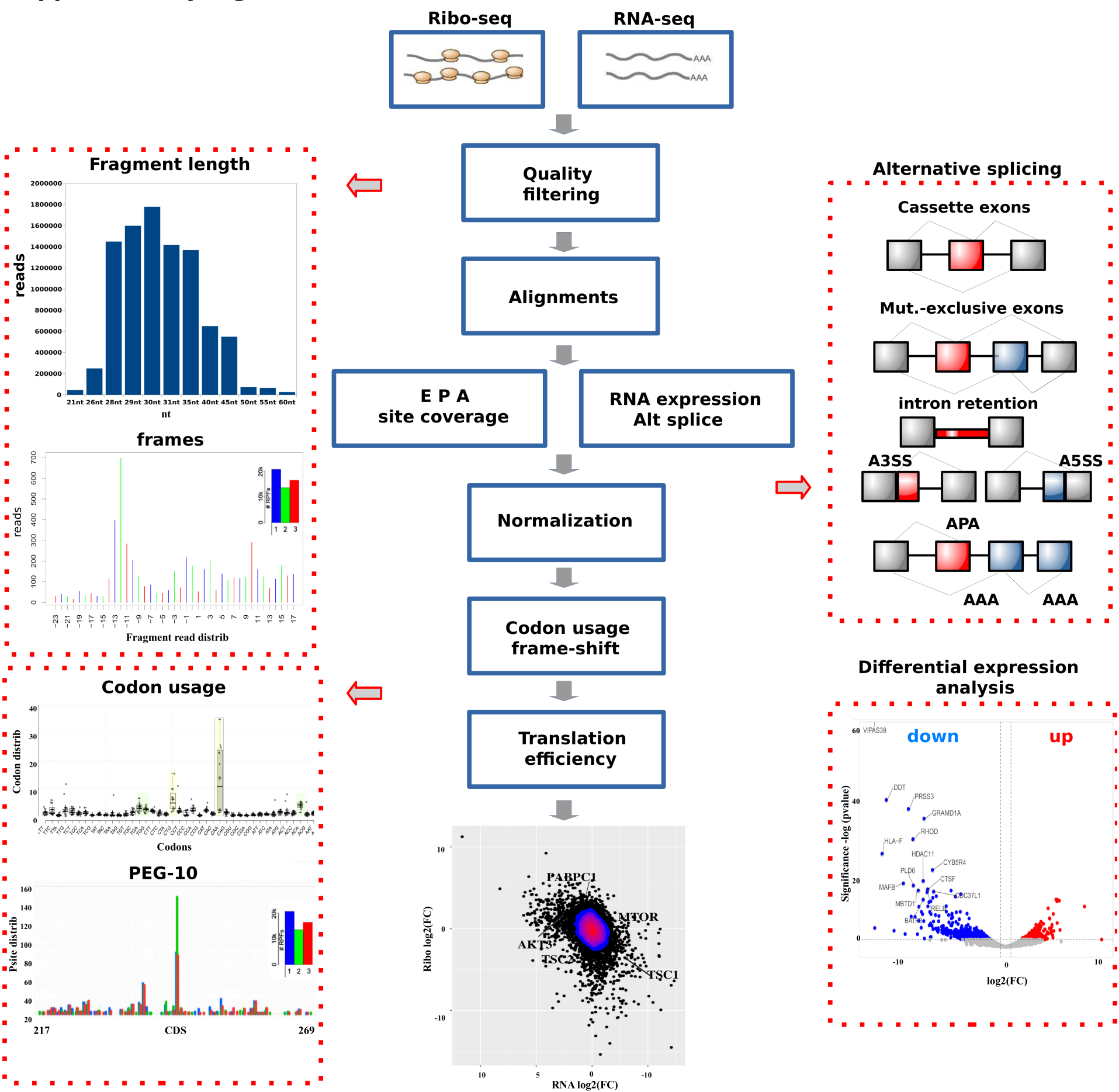

Supplement: Supplementary file 1 — Figure S1 [file ACEL-22-e13893-s003.pdf]

Supplementary Figure 3

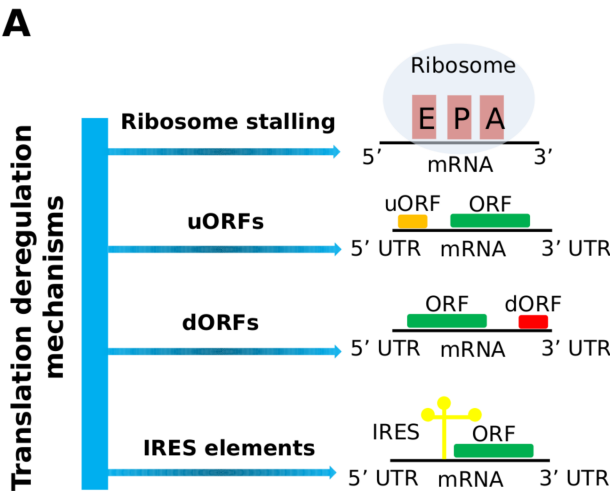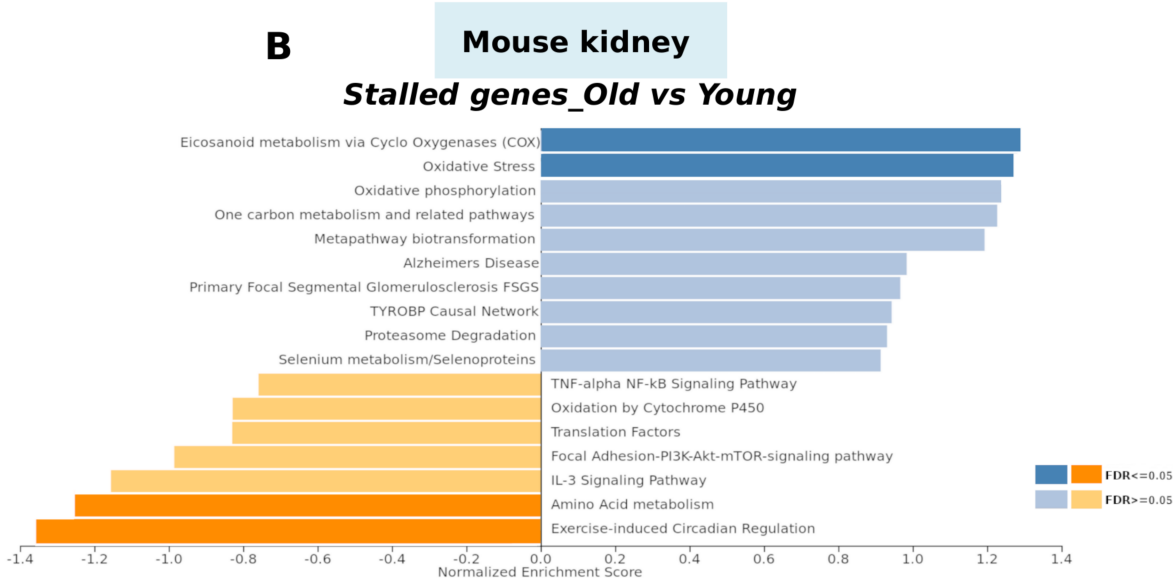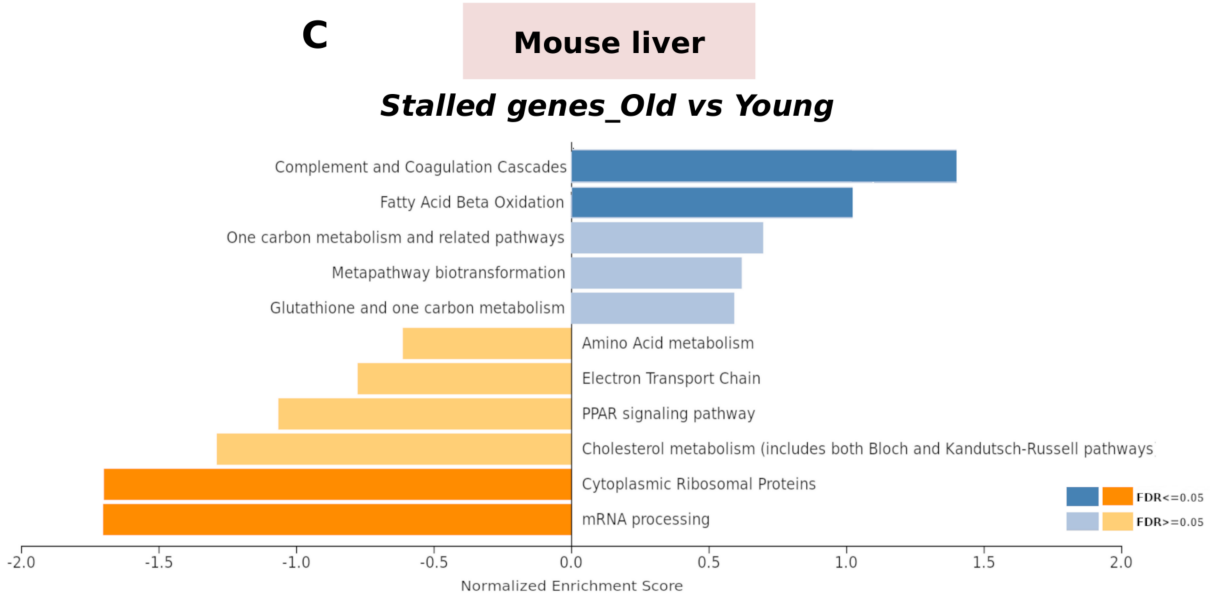

Supplement: Supplementary file 3 — Figure S3 [file ACEL-22-e13893-s007.pdf]

**Supplementary Figure 5**

**A**

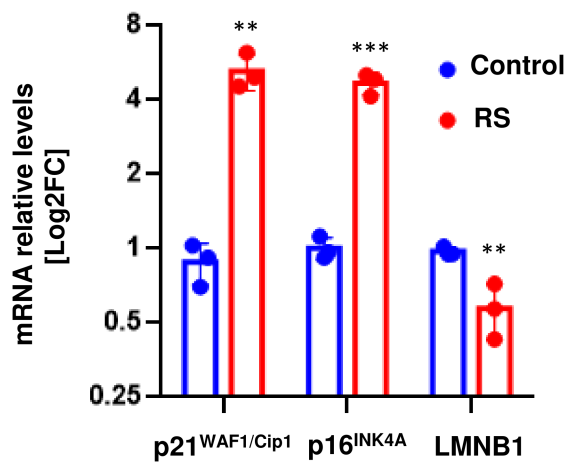

**B**

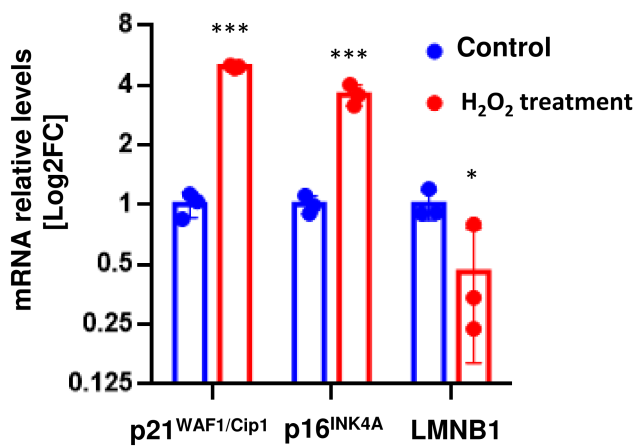

**C**

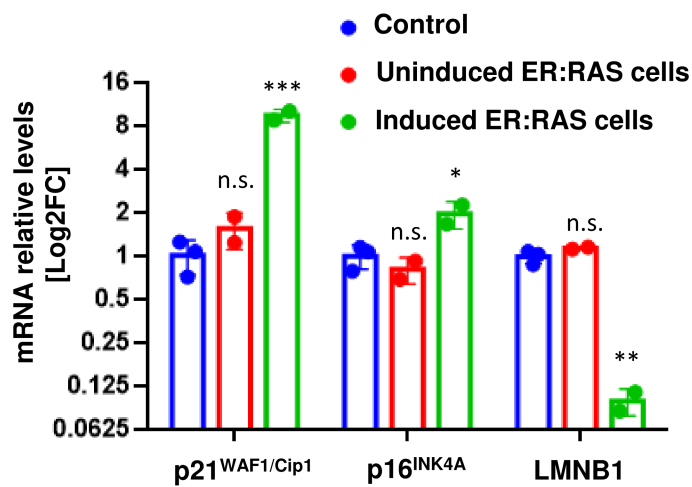

**D**

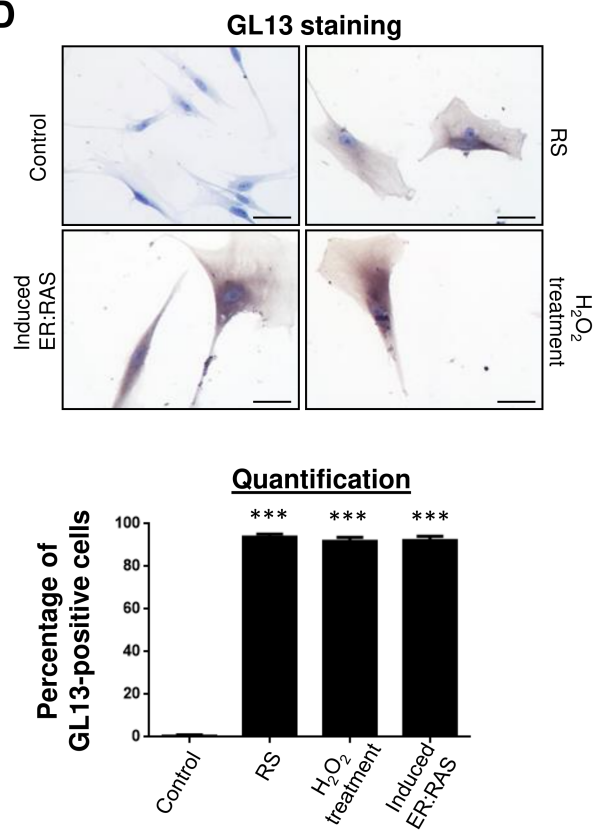

**E**

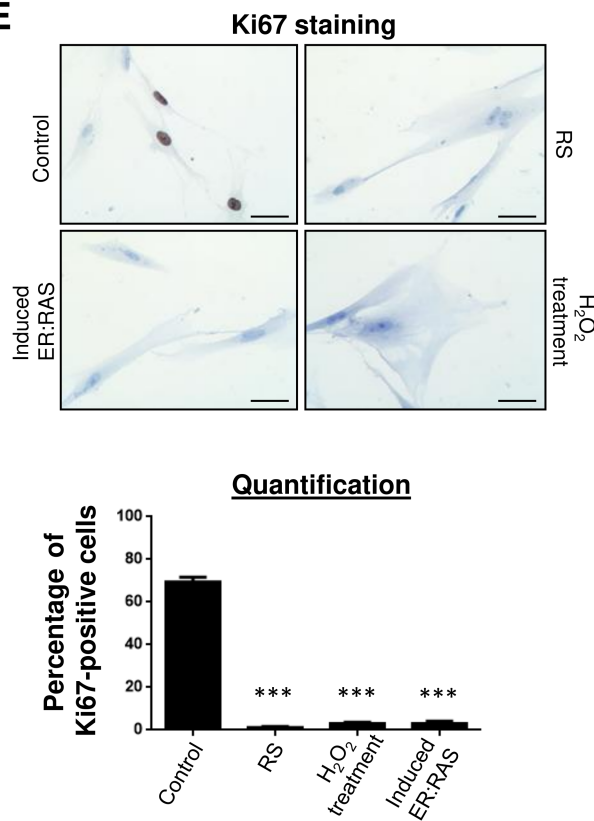

Supplement: Supplementary file 5 — Figure S5 [file ACEL-22-e13893-s011.pdf]

**A**

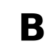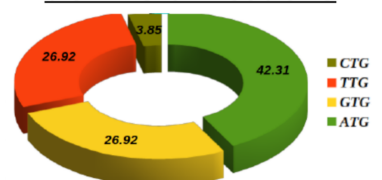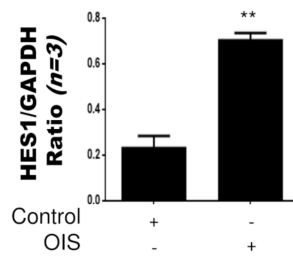

Supplement: Supplementary file 8 — Figure S8 [file ACEL-22-e13893-s009.pdf]
