# Supplementary material for: Decoding of translation‐regulating entities reveals heterogeneous translation deficiency patterns in cellular senescence
Source: Aging Cell. 2023 Aug 7;22(9):e13893. doi: 10.1111/acel.13893 (PMC10497830; doi:10.1111/acel.13893)

Supplementary Figure 2

A

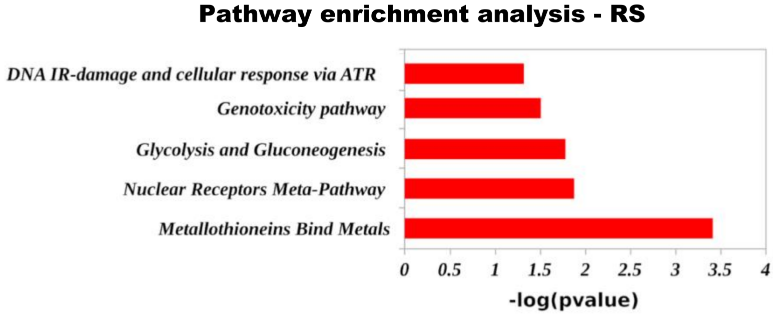

B

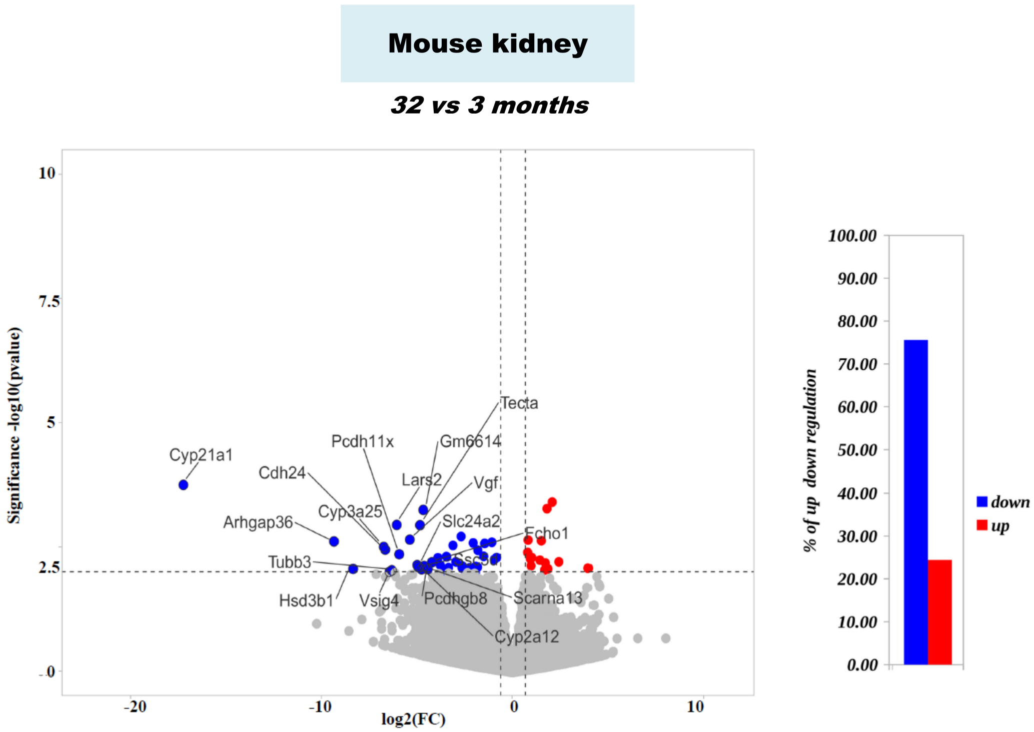

C

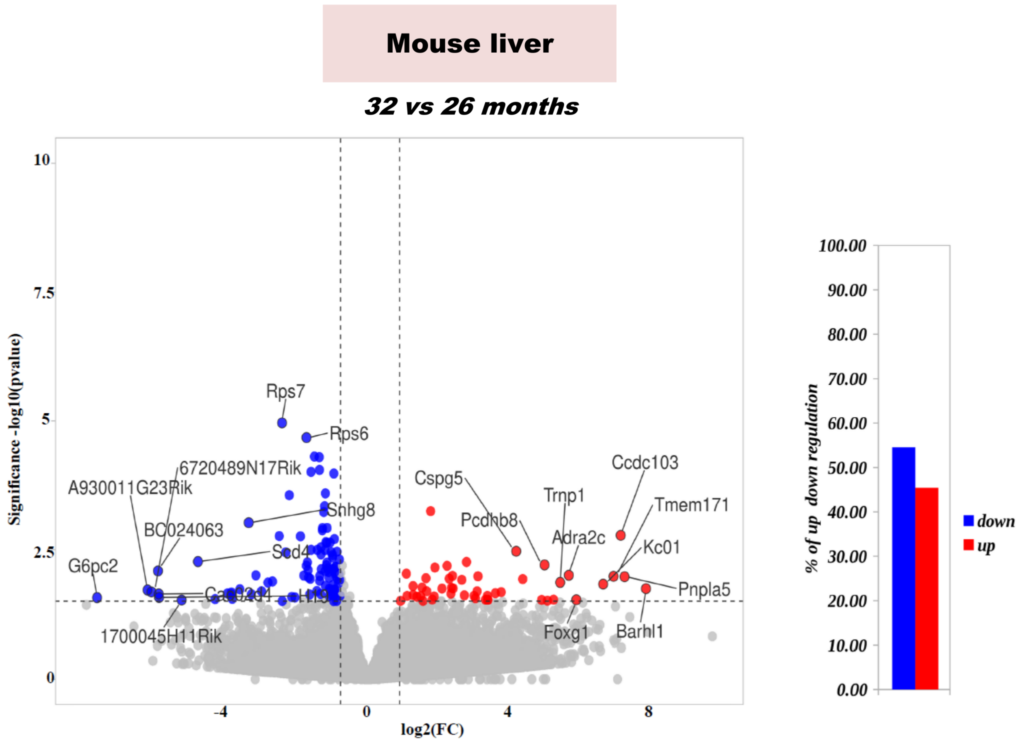

Supplement: Supplementary file 2 — Figure S2 [file ACEL-22-e13893-s010.pdf]
