# Supplementary material for: Decoding of translation‐regulating entities reveals heterogeneous translation deficiency patterns in cellular senescence
Source: Aging Cell. 2023 Aug 7;22(9):e13893. doi: 10.1111/acel.13893 (PMC10497830; doi:10.1111/acel.13893)

Supplementary Figure 7

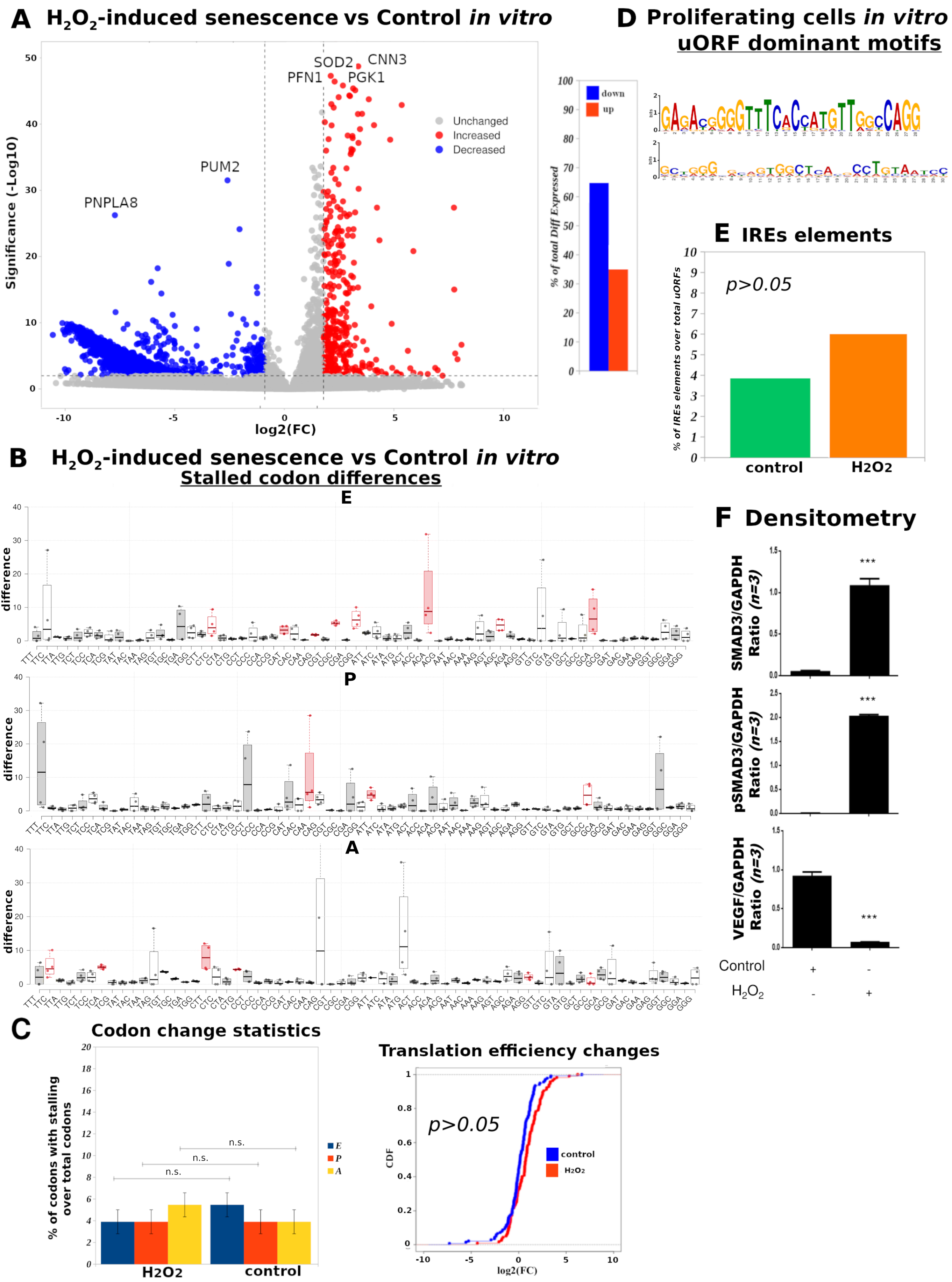

Supplement: Supplementary file 7 — Figure S7 [file ACEL-22-e13893-s006.pdf]
