# Supplementary material for: Decoding of translation‐regulating entities reveals heterogeneous translation deficiency patterns in cellular senescence
Source: Aging Cell. 2023 Aug 7;22(9):e13893. doi: 10.1111/acel.13893 (PMC10497830; doi:10.1111/acel.13893)
Supplement: Supplementary file 11 — Appendix S1 [file ACEL-22-e13893-s004.docx]

**Supplementary Figure 1. Computational pipeline for deciphering translation deregulation mechanisms upon replicative and stress-induced senescence.** Following ribosome profiling and RNA-seq data analysis, the consecutive steps of the workflow include quality and length filtering of the ribosome libraries, determination of the P-site offset and estimation of the read coverage, as well as examination of the ribosome periodicity for ORF, uORF, dORF identification and ribosome stalling. Moreover, RNA abundance and mRNA differential expression are examined via alternative splicing identification. Normalization of the ribosome coverage by using RNA-seq data enables recognition of translation efficiency changes and filtering of potential sequencing artifacts. Differential mRNA expression analysis is additionally used to construct pathway enrichment analyses based on genes and gene networks where mRNA translation deregulation occurs.

**Supplementary Figure 2. Translation deficiency changes in aged mouse tissue. A.** Pathway enrichment analysis on pooled data from W138 lung fibroblasts undergoing RS and aged mouse tissue (26 and 32 months old) versus proliferative and young (1 and 3 months old) counterparts, respectively. The analysis displayed increased expression of stress-related genes upon RS/aging. **B.** Volcano plots demonstrating genes with significantly decreased (blue) and increased (red) translation rate in kidney tissue from aged (32 months old) mice versus young counterparts (3 months old). Bar graphs indicate the percentage of significant (*p<0.05*) changes in translation efficiency. **C.** Same as B, for liver tissue from aged (32 months old) mice versus mice of advanced age (26 months old) indicating only minor translation efficiency changes. See also Table S2.

**Supplementary Figure 3. Stalled gene pathway enrichment analyses in mouse tissue. A.** Schematic depicting the most common translation deregulation mechanisms examined throughout the study; stalled ribosomes, uORFs, dORFs and IRES elements. **B.** Pathway enrichment analysis on stalled genes of aged (32 months old) versus young (3 months old) mouse kidney cells. Oxidative stress is one of the most upregulated cellular responses, while amino acid metabolism ranks among the most downregulated cellular responses, in line with the pronounced translation deficiency of senescent cells. **C.** Same as B, for aged (32 months old) versus young (3 months old) mouse liver cells. Amino acid metabolism and mRNA processing rank among the most downregulated cellular responses, in accordance with the impaired translation efficiency of senescent cells. FDR: False Discovery Rate.

**Supplementary Figure 4. Genes undergoing translation deregulation correlate with distinct cellular signaling in OIS cells. A.** Identification of uORF dominant motifs in proliferating BJ fibroblasts using the MEME motif finding platform. Those motifs appear to be G-enriched. **B.** Gene Set Enrichment Analysis (GSEA) using the Enrich platform for all transcripts where ribosome stalling and uORF/dORF patterns were observed in OIS versus control BJ fibroblast cells. All transcripts undergoing translation deregulation were pooled and overlapped with translation efficiency changes. Statistically significant genes were ranked based on the pathway enrichment and network analysis as performed with esyN and ConsensusPathDB (See Table S2 and Methods). **C.** mRNA levels of the transcripts in A (log2FC) plotted against translation efficiency changes (log2FC) when comparing OIS versus normal BJ fibroblasts. Transcripts of interest are marked using the R function “ggrepel”. **D.** Notch pathway positive regulators stand out from the analyses in B and C as they exhibit the clearest regulation pattern among all identified pathways, with a consistently higher translation level in OIS compared to control BJ fibroblasts.

**Supplementary Figure 5. *In vitro* induction of senescence in IMR-90 human lung fibroblasts.** Induction of cellular senescence was verified in IMR-90 cells via increased expression of *p21^WAF1/Cip1^* and *p16^INK4A^*, as well as decreased expression of *LMNB1*, assessed by q-PCR in **A.** control (proliferating) cells versus cells subjected to replication stress (RS), **B.** control cells versus H_2_O_2_-treated cells and **C.** control cells versus uninduced ER:RAS cells or ER:RAS cells induced to express the *RAS^G12V^* gene. **D.** GL13 immunocytochemichal staining of IMR-90 cells from indicated conditions to assess the presence of senescence via lipofuscin detection. Bar graphs provide quantification of GL13 signal intensity across conditions. **E.** Ki67 immunocytochemical staining of IMR-90 cells in D displays an inverse staining pattern to GL13. Bar graphs provide quantification of Ki67 signal intensity across conditions. **P<0.05*, ***P<0.01* and ****P<0.001*, of Student’s t-test; n.s., non-significant. Error bars indicate s.e.m. Data shown are representative of at least 3 biological experiments (n ≥ 3).

**Supplementary Figure 6. Replicative senescence induction and translation deficiency in IMR-90 cells. A.** Volcano plots of genes with significantly decreased (blue) and increased (red) translation rate in IMR-90 cells undergoing RS versus control. Bar graphs indicate the percentage of significant (*p<0.05*) changes in translation efficiency. See also Table S2. **B.** Left: Ribosome stalling in E-, P- or A- sites in IMR-90 cells under RS vs control. Red coloring in the box plot indicates codons where ribosomes are most stalled. Right: Bar graphs displaying percentage differences of stalled codons per ribosome site between RS and control. n.s., non-significant; Error bars indicate s.e.m.

**Supplementary Figure 7. Stress-induced senescence *in vitro* corroborates uORF implication in translation deregulation. A.** Volcano plots demonstrating genes with significantly decreased (blue) and increased (red) translation rate in H_2_O_2_-treated IMR-90 cells versus control. Bar graphs indicate the percentage of significant (*p<0.05*) changes in translation efficiency. See also Table S2. **B.** Ribosome stalling differences derived by comparing the normalized EPA coverage per codon for H_2_O_2_-treated IMR-90 cells versus control. Red coloring in the box plots indicates codons where ribosomes were most stalled. **C.** Left: Bar graphs depict non-significant changes in the percentages of stalled codons in E-, P- and A- ribosome sites between H_2_O_2_-treated IMR-90 cells and untreated counterparts. Right: CDF curve demonstrating an overall non-significant difference in translation efficiency between stalled codons in H_2_O_2_-treated cells versus control. **D.** Identification of uORF dominant motifs in proliferating IMR-90 fibroblasts using the MEME motif finding platform. Those motifs appear to be G-enriched, in line with published datasets. **E.** Bar graph showing the percentage (%) of IRES elements found in the indicated conditions. Differences were found insignificant (*p>0.05*). **F.** Densitometry providing quantification for Western blots in Fig. 5G. ****P<0.001*, of Student’s t-test; n.s., non-significant. Error bars indicate s.e.m. Data shown are representative of at least 3 biological experiments (n ≥ 3).

**Supplementary Figure 8. *RAS^G12V^*-induced senescence in IMR-90 cells recapitulates enrichment of all translation deregulation mechanisms *in vitro*. A.** Volcano plots of genes with significantly decreased (blue) and increased (red) translation rate in human primary IMR-90 cells undergoing OIS versus control. Bar graphs indicate the percentage of significant (*p<0.05*) changes in translation efficiency. See also Table S2. **B.** Ribosome stalling differences in OIS IMR-90 versus control cells derived by comparing the normalized EPA coverage per codon. Red coloring in the box plots indicates codons where ribosomes are most stalled. **C.**The CDF plot of the transcripts where stalling is observed shows a significant drop (*p<0.05*) in translation efficiency in OIS. **D.** Distribution of uORF start codons in OIS IMR-90 cells. **E.** Distribution of dORF start codons in OIS IMR-90 cells. **F.** Pathway enrichment analysis for genes regulated by ribosome stalling and uORFs/dORFs using the WebGestalt platform. Red: significantly upregulated pathways (*p<0.05*); Blue: significantly downregulated pathways (*p<0.05*); Orange: marginally upregulated pathways (*p≥0.05*). **G.** Densitometry providing quantification for Western blots in Fig. 5O. ***P<0.01*, of Student’s t-test. Error bars indicate s.e.m. Data shown are representative of at least 3 biological experiments (n ≥ 3).

**Supplementary Table 1.** List of all published datasets used in this study.

**Supplementary Table 2.** Raw data resulting from the ribosome stalling, uORF/dORF, IRES identification and gene prioritization analyses carried out in this study for all examined senescence types versus respective controls.
